# Fate-mapping infiltrating monocytes following experimental myocardial infarction reveals differentiation trajectories in the infarcted heart

**DOI:** 10.1172/JCI189684

**Published:** 2026-07-07

**Authors:** Andrew L. Koenig, Farid F. Kadyrov, Junedh M. Amrute, Steven Yang, Carla J. Weinheimer, Jessica M. Nigro, Attila Kovacs, Wenjun Li, Gabriella B. Smith, Lance Yeh, Daniel Kreisel, Kory J. Lavine

**Affiliations:** 1 Center for Cardiovascular Research, Division of Cardiology, Department of Medicine,; 2 Department of Surgery,; 3 Department of Pathology and Immunology, and; 4 Department of Developmental Biology, Washington University School of Medicine, Saint Louis, Missouri, USA.

**Keywords:** Cardiology, Immunology, Cellular immune response, Heart failure, Macrophages

## Abstract

Inflammation contributes to the pathogenesis of myocardial infarction and heart failure and represents a viable therapeutic target. Monocytes and their progeny are highly abundant and display striking functional diversity, serving as key determinants of myocardial inflammation and tissue repair. Much remains to be learned regarding mechanisms and signaling events that instruct monocyte fate decisions. We devised a genetic lineage tracing strategy using *Ccr2^crERT2^Rosa26^LSL–tdTomato^* mice in combination with single cell RNA-seq to map the differentiation trajectories of monocytes that infiltrate the heart after reperfused myocardial infarction. Monocytes were recruited to the heart early after injury and gave rise to transcriptionally distinct and spatially restricted macrophage and dendritic cell–like subsets that were specified prior to extravasation and chronically persisted within the myocardium. Pseudotime analysis predicted 2 differentiation trajectories of monocyte-derived macrophages that are partitioned into the border and infarct zones, respectively. Among these trajectories, we demonstrated that macrophages expressing a type I interferon–responsive signature were an intermediate population that gave rise to MHC-II^hi^ macrophages, were localized within the border zone, induce regulatory T cells, and promote myocardial protection. Collectively, these data uncover complexities of monocyte differentiation in the infarcted heart and suggest that modulating monocyte fate decisions may have clinical implications.

## Abstract 2

In myocardial infarction and heart failure, monocytes differentiate into distinct macrophage subsets, influencing inflammation and tissue repair and offering potential therapeutic targets through fate modulation.

## Introduction

Myocardial infarction (MI) remains a leading cause of death and major cause of heart failure worldwide (1, 2). It is well established that ischemic damage to the heart promotes myocardial inflammation by triggering recruitment of peripheral leukocytes that impart a second hit to the heart and contribute to additional cardiomyocyte loss, fibrosis, adverse left ventricular (LV) remodeling, and impaired cardiac function. Among recruited leukocytes, monocytes and macrophages represent the most abundant immune cells to accumulate within the infarcted and failing heart (3).

Within the past decade, it has been established that cardiac macrophages are functionally heterogeneous and can be broadly divided into 2 populations distinguished by expression of C-C chemokine receptor 2 (CCR2). CCR2^–^ macrophages are an embryonically derived, long-lived population that comprises the major macrophage subset in the healthy heart. CCR2^–^ macrophages are self replenishing, express conserved markers of tissue resident macrophages, and function to orchestrate cardiac development, homeostasis, adaptation, and repair (4–9). In contrast, CCR2^+^ macrophages are an inflammatory population derived from recruited LY6C^hi^ monocytes that accumulate within the injured and chronically failing heart. CCR2^+^ macrophages are associated with and causally linked to heart failure, as they contribute to damaging inflammation, myocardial fibrosis, and adverse LV remodeling (6, 10–17).

Recent studies from our group have leveraged single-cell transcriptomics in both mice and humans to demonstrate additional complexity within cardiac monocyte and macrophage populations (3, 18–22). These studies have uncovered marked diversity among CCR2^+^ monocyte-derived macrophages that infiltrate the infarcted heart. While these findings have suggested that monocytes may differentiate into functional diverse subpopulations of CCR2^+^ macrophages, little is known regarding their spatial-temporal dynamics, trajectories, and mechanisms by which monocytes differentiate into these populations.

Here, we construct a map of monocyte differentiation trajectories in reperfused myocardial infarction (MI) using single-cell RNA-seq. We demonstrate that monocytes are recruited to the heart early after MI and give rise to transcriptionally distinct and spatially restricted macrophage and dendritic cell–like subsets that chronically persist within the myocardium. Monocyte-derived cells adopt macrophage and dendritic cell fates as they extravasate out of the vascular compartment and dynamically shift their phenotype over time. We leveraged pseudotime analysis and spatial transcriptomics to predict spatially restricted monocyte differentiation trajectories. Finally, we provide experimental validation and show that macrophages expressing a type I IFN–responsive signature are an intermediate population that gives rise to MHC-II^hi^ macrophages and that this trajectory confers protection following myocardial injury. These findings highlight unrecognized complexities of monocyte differentiation and suggest that modulating monocyte fate decisions may have clinical implications.

## Results

### Monocytes are recruited to the heart early after MI and their progeny chronically persist within the myocardium.

**Figure 1:**
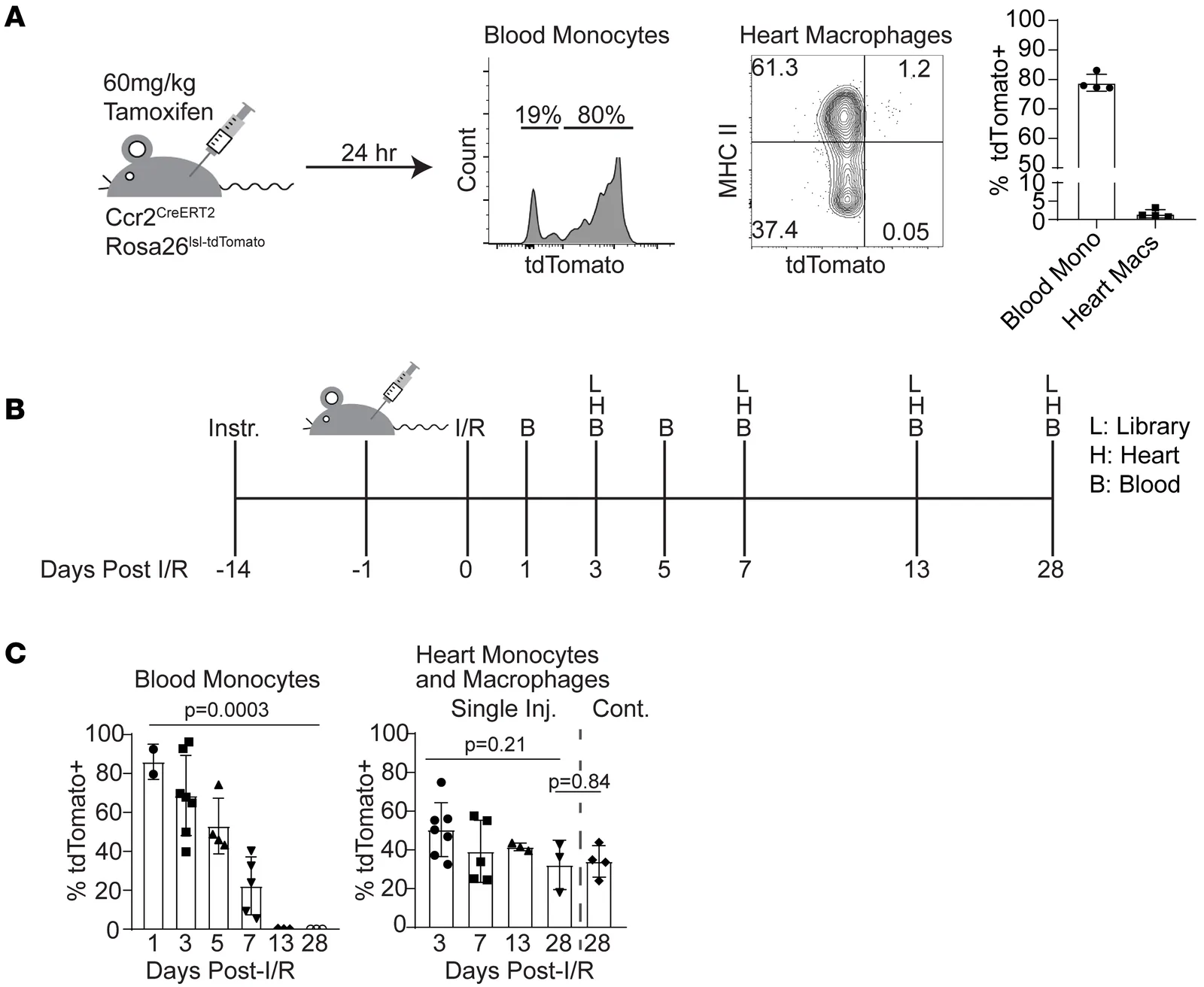
Recruited monocyte-derived populations persist in the heart after injury. (**A**) Representative flow cytometry plots demonstrating that a single dose of tamoxifen administered to *Ccr2Cre^ERT2^Rosa26^tdTomato^* mice results in 80% labeling efficiency in monocytes in the blood, while resident cardiac macrophages are not labeled. (**B**) Schematic of experimental strategy for monocyte lineage tracing after MI. (**C**) Bar graphs demonstrating tdTomato labeling of blood monocytes (left) and cardiac monocyte and macrophages. Single inj., single dose labeling; cont, continuous dose labeling. Brown-Forsythe 1-way ANOVA.

To trace the fates of monocytes and their progeny after MI, we subjected *Ccr2^CreERT2^Rosa26^LSL–tdTomato^* mice to closed-chest ischemia reperfusion injury to model reperfused MI (23–25). *Ccr2^CreERT2^* mice harbor a tamoxifen-inducible CRE recombinase inserted into the 3’ UTR of the *Ccr2* locus. We optimized a tamoxifen induction strategy to exclusively label circulating monocytes and not cardiac macrophages. A single injection of tamoxifen (60 mg/kg, IP) into naive mice achieved selective tdTomato labeling of LY6C^hi^ monocytes in the blood at greater than 80% efficiency 24 hours after injection. Cardiac macrophages did not express tdTomato at day 1 or day 7 after injection (Figure 1A, Supplemental Figure 1; supplemental material available online with this article; https://doi.org/10.1172/JCI189684DS1). We also did not detect substantial tdTomato labeling of neutrophils or lymphocytes in the blood or their progenitors within the bone marrow compartment (Supplemental Figure 2). As such, this strategy allows lineage tracing of infiltrating monocytes and their progeny independent of resident macrophages.

**Figure 2:**
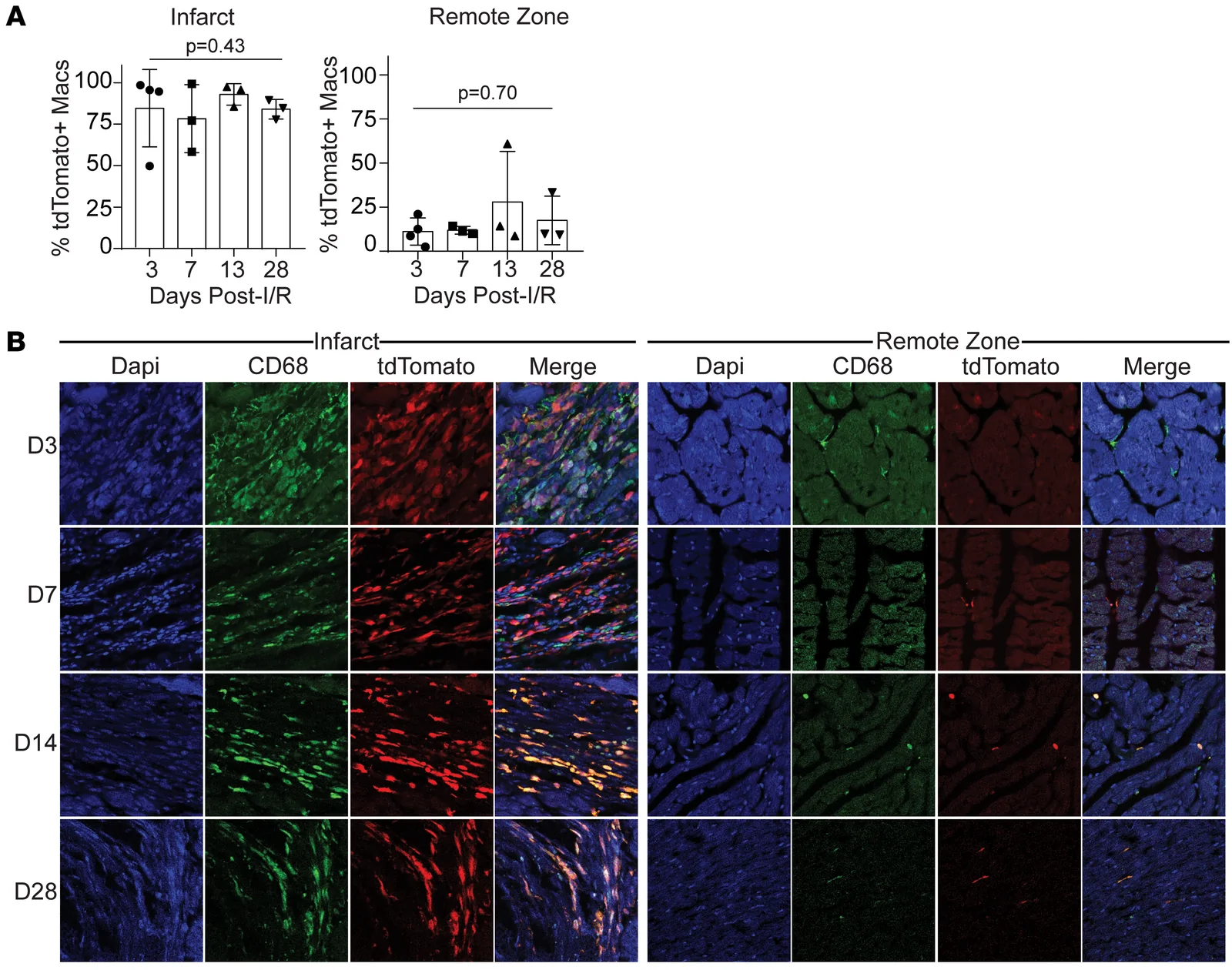
Recruited monocyte-derived populations are concentrated within the infarcted area. (**A**) Quantification of tdTomato+ macrophages relative to total CD68-positive macrophages measured by IHC. (**B**) Representative IHC images. Magnification ×20. Each data point represents an individual animal. Brown-Forsythe 1-way ANOVA.

We then applied this labeling strategy to mice subjected to closed-chest ischemia reperfusion injury. Tamoxifen was injected 1 day before injury. Blood was collected from mice at days 1, 3, 5, 7, 13, and 28 after injury and hearts were collected at days 3, 7, 13, and 28 after injury (Figure 1B). Flow cytometry analysis demonstrated greater than 80% tdTomato labeling efficiency in LY6C^hi^ monocytes in the blood 1 day after MI. The percent of LY6C^hi^ monocytes labeled progressively decreased throughout the first 7 days after MI and was absent thereafter. Intriguingly, the progeny of monocytes that entered the heart during this labeling window persisted for at least 28 days after MI. Continuous labeling of monocytes by tamoxifen injection (60 mg/kg IP, every 3 days) did not demonstrate additional labeling of cardiac macrophages (Figure 1C). Labeling of macrophages within the heart was validated at each time point by visualization of tdTomato and immunostaining for CD68 (Figure 2, A and B). These findings indicate that monocytes enter the heart predominately within the first 7 days after MI and their progeny, including monocyte-derived macrophages, persist within the myocardium for at least 28 days after MI.

### Macrophage states dynamically shift over time after injury.

**Figure 3:**
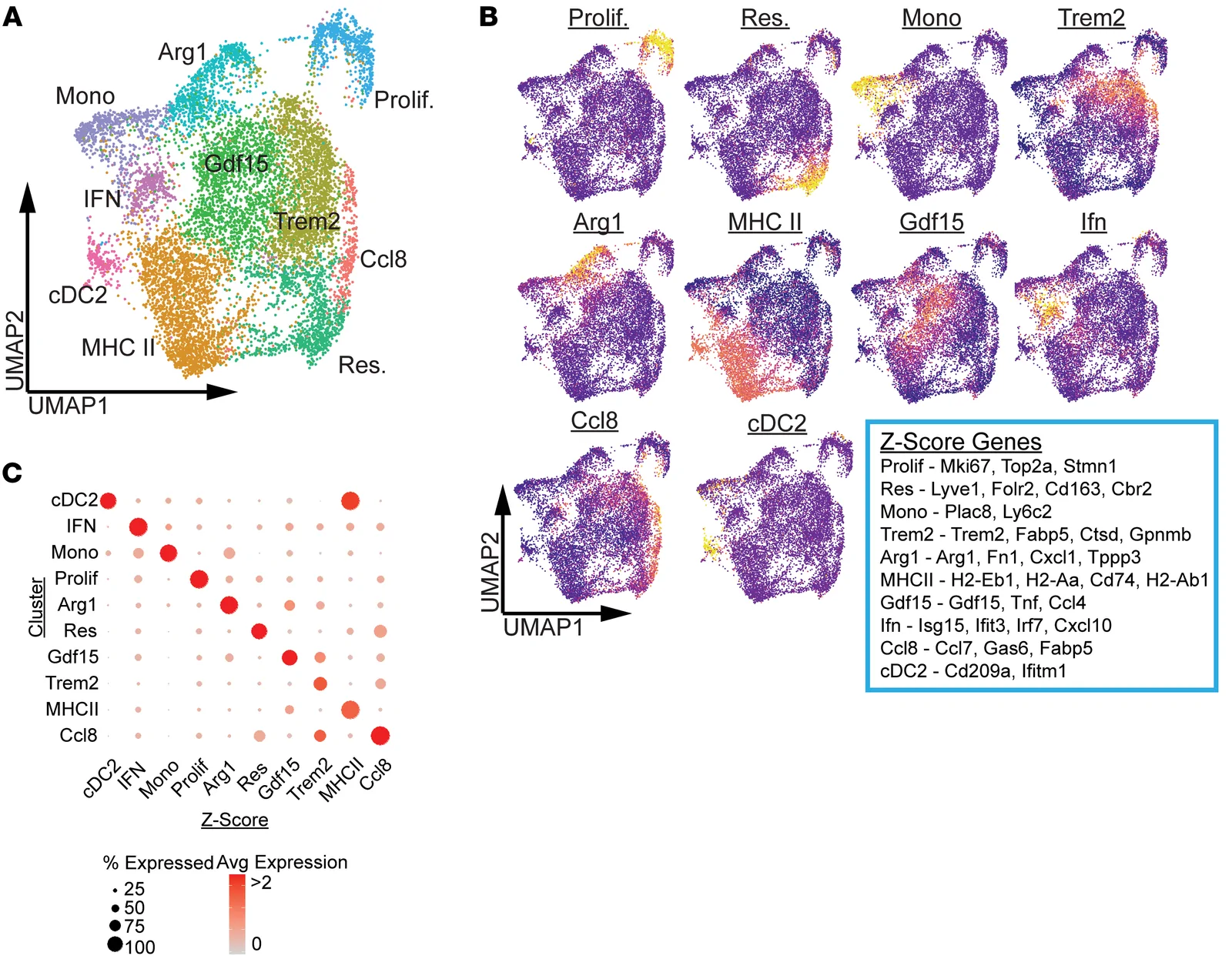
Recruited monocyte-derived populations are transcriptionally diverse. (**A**) Clustered UMAP projection of combined monocyte lineage tracing single-cell libraries from each time point. (**B**) Feature plots of calculated z-scores for each cluster. Blue box, list of genes in each z-score. (**C**) Dot plot of z-scores by cluster.

**Figure 4:**
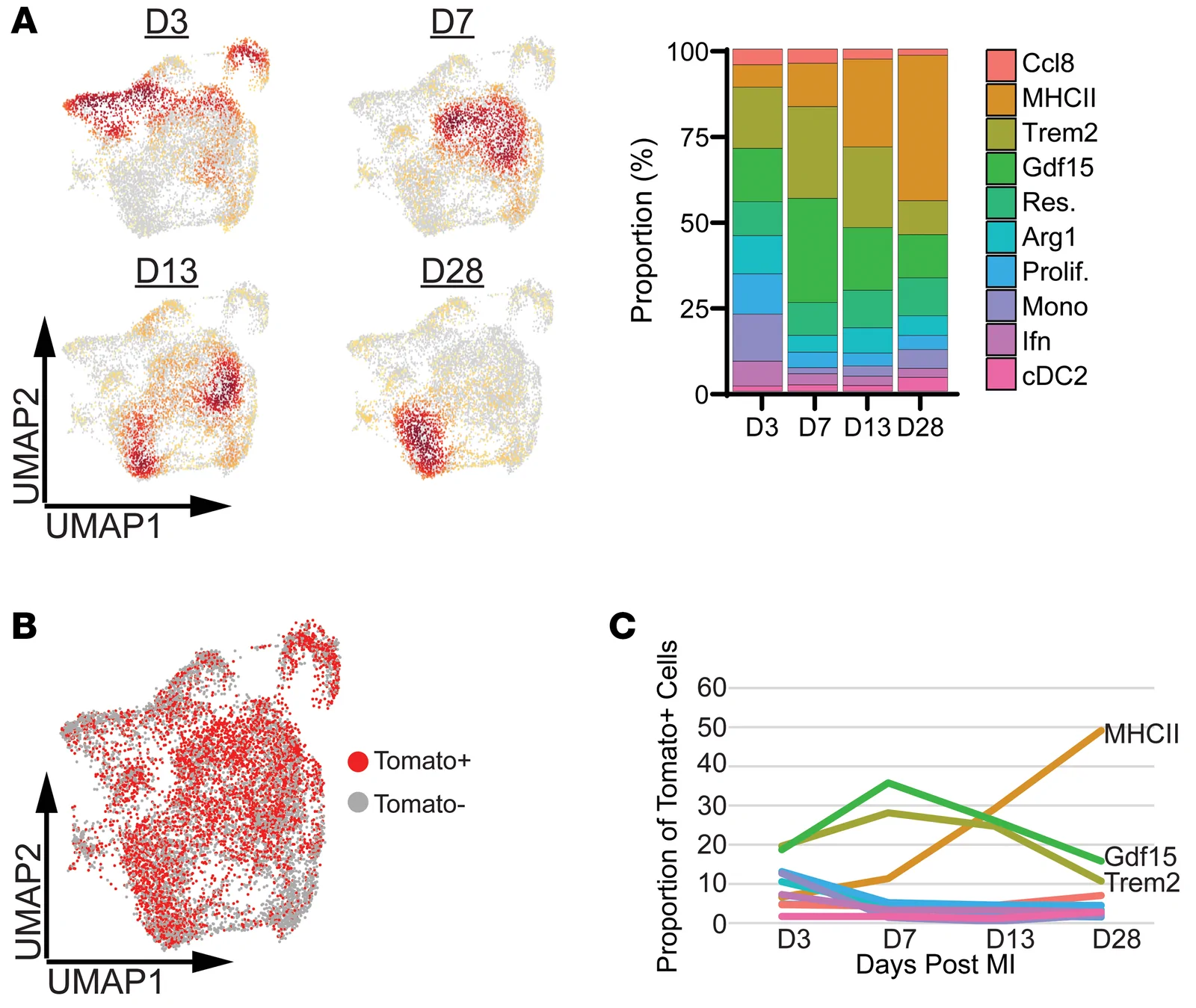
Monocyte-derived populations dynamically shift as a function of time after MI. (**A**) Gaussian kernel plots indicating cell density at each time point after MI (left) and proportion of cells in each cluster split by time point (right). (**B**) UMAP plot of Tomato+ cells. (**C**) Line graph of proportion of tdTomato+ cells in each cluster across time points.

To delineate the temporal dynamics of recruited monocytes and their progeny, we performed single-cell RNA-seq of monocytes and macrophages sorted from *Ccr2^CreERT2^Rosa26^LSL–tdTomato^* hearts collected at days 3, 7, 13, and 28 after MI (Figure 1B and Supplemental Figure 3A). Libraries were constructed for each time point, sequenced, aligned using Cell Ranger, and then integrated, clustered, and analyzed using Seurat (26, 27). We identified 10 transcriptionally distinct cell clusters: monocytes, dendritic cells, and 8 macrophage states (Figure 3A). We performed differential expression analysis and constructed z-score signatures to support that the observed clusters were transcriptionally distinct (Figure 3, B and C, Supplemental Figure 4). When cells were compared across time points, it was evident that macrophage states markedly shifted over time. At 3 days after MI, the predominant populations within the heart consisted of monocytes, proliferating macrophages, *Arg1^+^* macrophages, type-I interferon (IFN) activated macrophages, and *Trem2^+^* macrophages. Through days 7 and 13 after MI, we observed decreases in frequency of monocytes, proliferating cells, *Arg1^+^* macrophages, and type I IFN–activated macrophages. *Trem2^+^* macrophages and *Gdf15^+^* macrophages expanded during this time interval. By day 28 after MI, macrophages had transitioned to a predominantly MHCII^hi^ state (Figure 4A).

We additionally performed custom alignment of the libraries to the tdTomato coding sequence and observed that all identified clusters were labeled by expression of tdTomato (Figure 4B). Interestingly, a proportion of macrophages within the resident cluster were also labeled by tdTomato, indicating that recruited macrophages have the potential to adopt a resident-like fate consistent with our prior findings (28). When comparing differential expression of tdTomato^–^ and tdTomato^+^ resident macrophages, we identified that monocyte-derived resident-like (tdTomato^+^) macrophages demonstrate reduced expression of the tissue resident macrophage markers *Folr2* and *Lyve1* and enriched expression of *Ccl2, Ccl7,* and *Ccl12* compared with cardiac resident (tdTomato^–^) macrophages (Supplemental Figure 4).

We next examined the dynamics of monocyte-derived cells as a function of time and found that tdTomato^+^ cells were substantially distributed across cell states at day 3 after MI. At days 7 and 14 after MI, the number of tdTomato^+^ monocytes, *Arg1^+^* macrophages, type I IFN–activated macrophages, and proliferating macrophages declined, and *Gdf15^+^* and *Trem2^+^* macrophages progressively increased. At day 28 after MI, the majority of tdTomato^+^ cells were MHCII^hi^ macrophages (Figure 4C). Together, these results demonstrate that monocyte recruitment occurs within the first week after MI, their progeny persist within the heart at chronic stages, and monocytes display substantial plasticity differentiating into multiple transcriptionally distinct subsets that dynamically shift over time.

### Monocyte-derived macrophage and dendritic cell–like states exist outside of the cardiac resident macrophage compartment.

**Figure 5:**
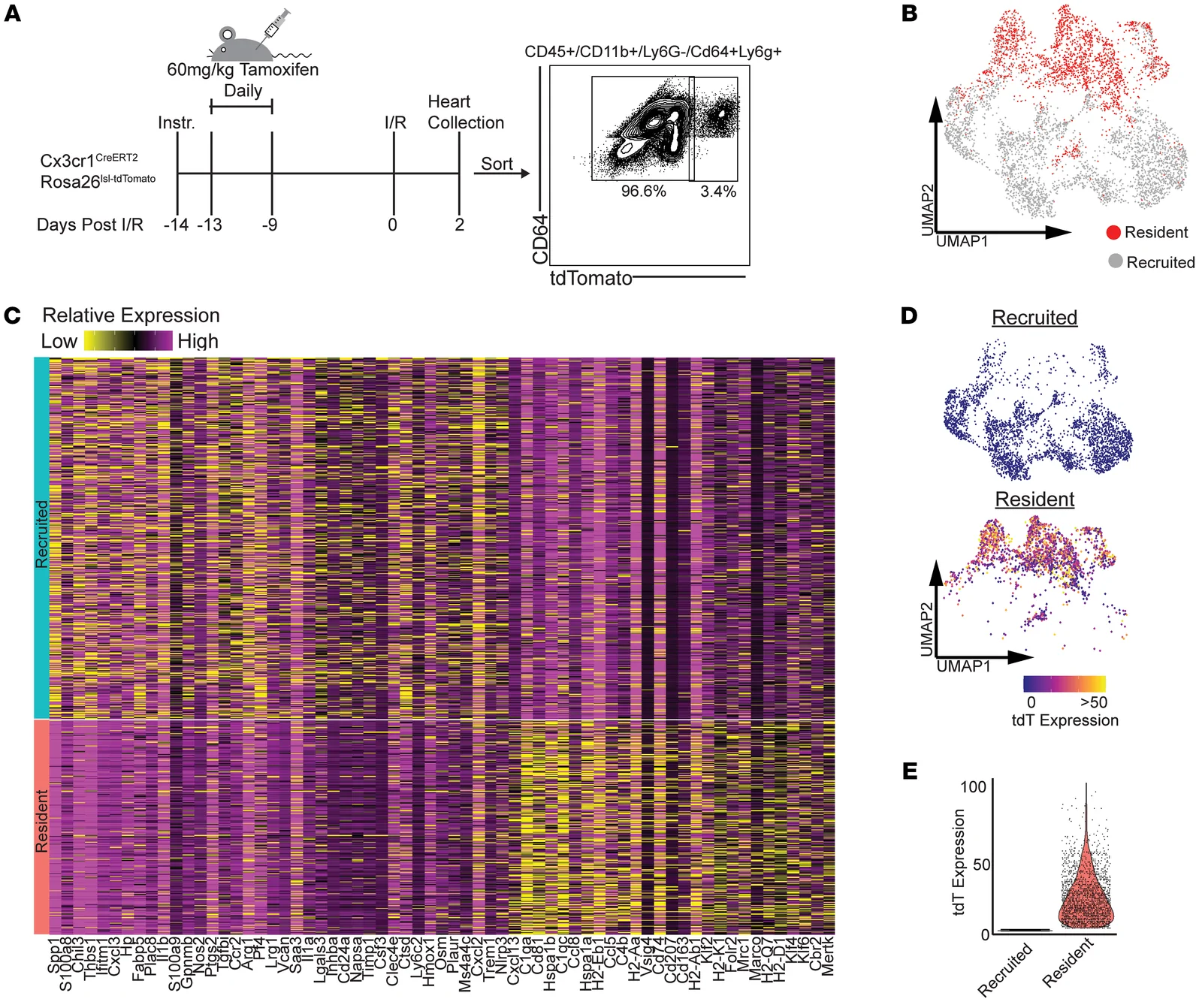
Monocyte-derived populations exist independent of cardiac resident macrophages. (**A**) Schematic of experimental strategy for resident macrophage lineage tracing (left) and FACS gating strategy for constructing tdTomato+ (resident) and tdTomato– (recruited) libraries. (**B**) Combined UMAP plot of resident and recruited libraries. Red indicates cells derived from cardiac resident macrophages. (**C**) Heatmap of genes differentially expressed in cells derived from either cardiac resident macrophages or recruited monocytes. (**D**) Feature plot of tdTomato expression split by library. (**E**) Violin plot of tdTomato expression split by library.

We utilized *Cx3cr1^CreERT2^Rosa26^LSL–tdTomato^* mice to evaluate the contribution of resident macrophages to the diverse subsets of macrophage states that emerge following MI. Utilizing a pulse-chase strategy, we injected mice with tamoxifen (60 mg/kg daily X5 days) 2 weeks before ischemia reperfusion injury (Figure 3A). This strategy specifically labels long-lived resident macrophages with tdTomato. Monocytes are not labeled, as they are replenished by unlabeled monocytes during the chase period (13). Macrophages and monocytes were sorted via FACS into tdTomato^+^ (resident) and tdTomato^–^ (recruited) libraries for single-cell RNA-seq (Figure 5A and Supplemental Figure 3B). We observed that cardiac resident macrophages clustered independently of recruited macrophages and displayed distinct transcriptional profiles. Differential gene expression analysis demonstrated that resident and recruited macrophages express distinct transcriptional signatures. Many of the genes enriched in monocyte-derived macrophages were markers of the diverse populations seen in *Ccr2^CreERT2^* lineage tracing. Conversely, classical markers of tissue resident macrophages were enriched within the resident cardiac macrophage library (*Cd163, Mrc1, Cd207, Vsig4, Folr2, Cbr2*). The resident macrophage library is also enriched for components of the complement pathway (*C1qa, C1qc, C4b*). This is consistent with the role of resident macrophages initiating the inflammatory cascade after injury and the role of the complement pathway in recruiting monocytes (29, 30). At this earlier time point after injury, expression of MHCII genes appear to be enriched primarily in the resident macrophage library. In interpreting this finding, it should be noted that very few recruited MHCII^hi^ macrophages exist within the heart at this early time point. Thus, these data are most consistent with studies demonstrating existence of MHCII^hi^ and MHCII^lo^ resident macrophages (Figure 5, B and C) (31). To validate that the resident macrophage lineage tracing labeling was efficient, we aligned the single cell libraries to the tdTomato coding sequence and found that tdTomato was exclusively expressed in cells found within the resident macrophage library (Figure 5, D and E). These data indicate that resident macrophages do not contribute to the diverse macrophage subsets marked by *Ccr2-CreERT2* lineage tracing, consistent with their origin from infiltrating monocytes.

### Macrophage states identified after MI in mice are conserved in human heart failure.

**Figure 6:**
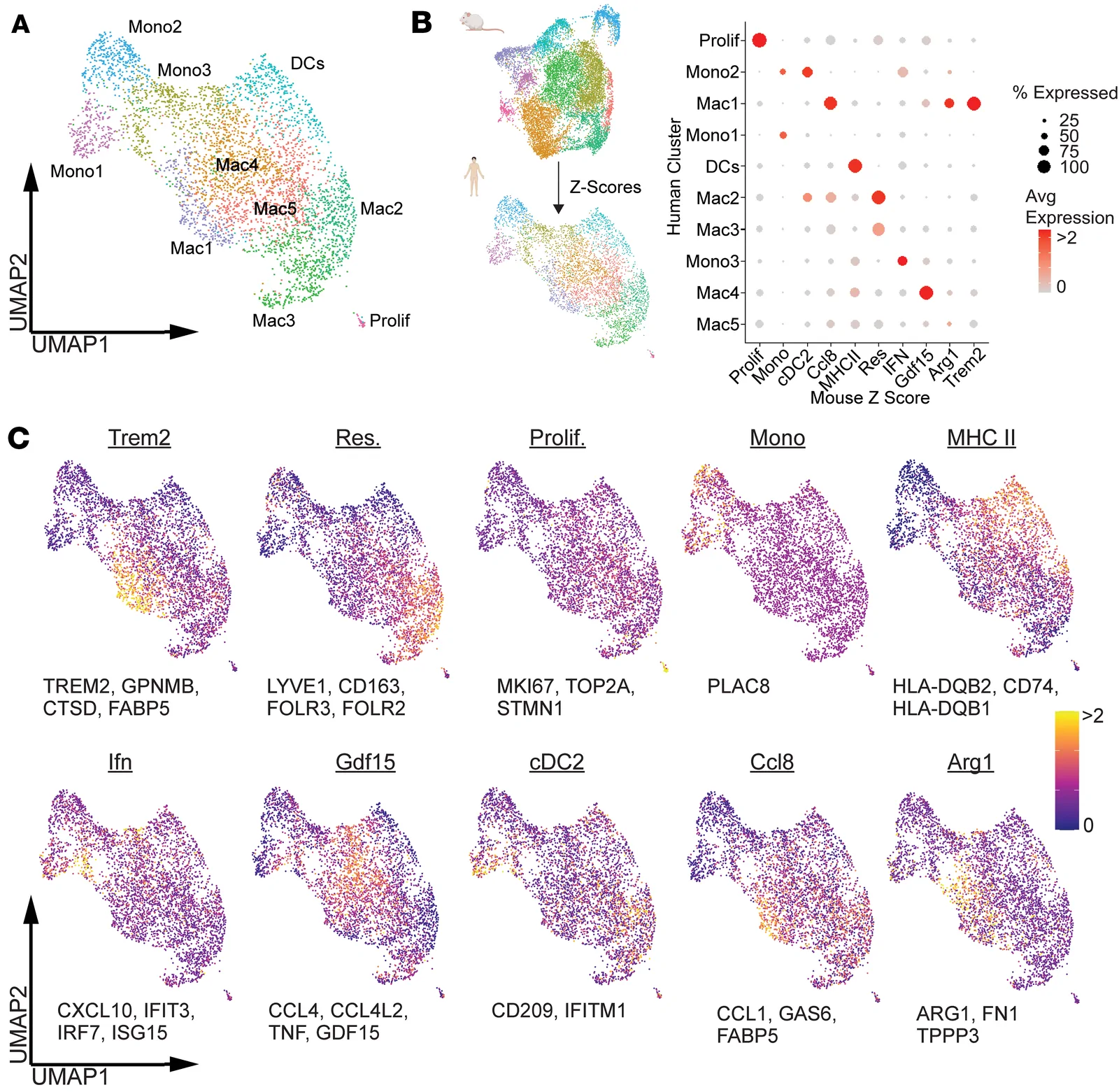
Monocyte-derived populations are conserved between mice and humans. (**A**) UMAP plot of monocytes, macrophages, and dendritic cells from human donor (*n* = 2) and heart failure samples (*n* = 5). (**B**)Schematic of analysis strategy (left) and dot plot of denoting the expression of genes enriched in each mouse monocyte, macrophage, and dendritic cell population (displayed as a z-score) in corresponding human monocyte, macrophage, and dendritic cell populations. Genes with mouse and human homologues are included in this analysis. (**C**) UMAP feature plots of each mouse z-score signature in the human object.

We utilized human single-cell RNA-seq data obtained from a prior study to determine if the observed monocyte, macrophage, and dendritic cell populations are conserved in the human heart. Transcription signatures for each mouse cluster were converted to their corresponding human homologues. Z-scores for these marker genes were calculated across human cardiac monocytes, macrophages, and dendritic cell clusters (Figure 6, A–C) (32). This analysis demonstrated that transcriptional signatures enriched in each mouse monocyte, macrophage, and dendritic cell cluster were conserved in corresponding populations of monocytes, macrophages, and dendritic cells found in the human heart, indicating that mice may serve as an appropriate model to investigate human monocyte differentiation.

**Figure 7:**
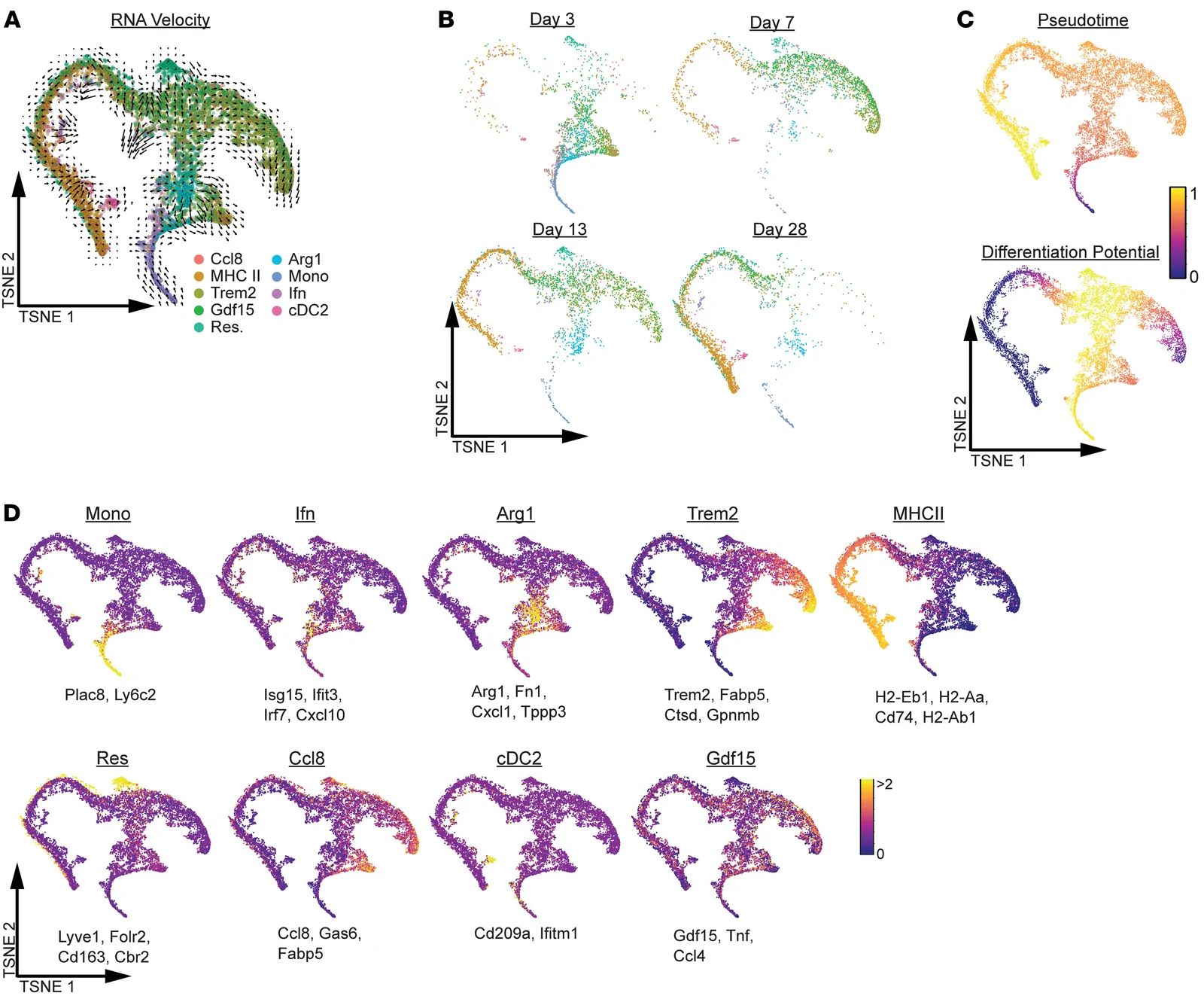
Monocyte-derived populations are temporally distinct. (**A** and **B**) Palantir tSNE projection of combined time points labeled by cluster with superimposed RNA velocity information (**A**) and split by time point (**B**). (**C**) Palantir tSNE projection labeled by pseudotime and differentiation potential. (**D**) Feature plots of monocyte, macrophage, and dependent cell populations z-scores overlayed on the tSNE projection.

Arg1^+^ and IFN-activated macrophages are predicted as early transient macrophage states. We utilized scVelo and Velocyto.R to perform RNA Velocity lineage tracing analysis (33, 34). We presented the data using t-distributed Stochastic Neighbor Embedding (t-SNE) projections split by time point after MI (Figure 7, A and B). RNA Velocity suggested that monocytes differentiate into *Arg1^+^* and IFN-activated macrophage populations as early intermediates, which subsequently differentiate to terminal *Trem2^+^* and MHCII^hi^ macrophages, respectively (Figure 7A). Splitting the t-SNE plot by time point after MI revealed 2 distinct populations of *Trem2^+^* macrophages, one arising at day 3 and a second evident beginning at day 7. The early *Trem2^+^* macrophage population expressed a distinct transcriptional signature that includes *Arg1* and *Spp1*. The later *Trem2^+^* macrophage population displayed enriched expression of *Ninj1, Creg1,* and *Gstm1* (Figure 7A and Supplemental Figure 5A). Each of these *Trem2^+^* macrophage populations was also observed in an independent trajectory analysis using Palantir (35). Palantir predicted that cDC2s, *Trem2*^+^ macrophages, and MHCII^hi^ macrophages were terminal cell states, and that type I IFN–activated macrophages and *Arg1^+^* macrophages represented intermediate states of differentiation based on entropy and pseudotime scores (Figure 7C). Plotting z-scores of each population also demonstrated increasing expression of terminal state markers further down the differentiation trajectories (Figure 7D). Consistent with this framework, monocytes, *Arg1^+^* macrophages, and type I interferon activated macrophages were enriched at early time points post-MI and positioned directly adjacent to monocytes in pseudotime vs. entropy waterfall plots (Supplemental Figure 5B).

**Figure 8:**
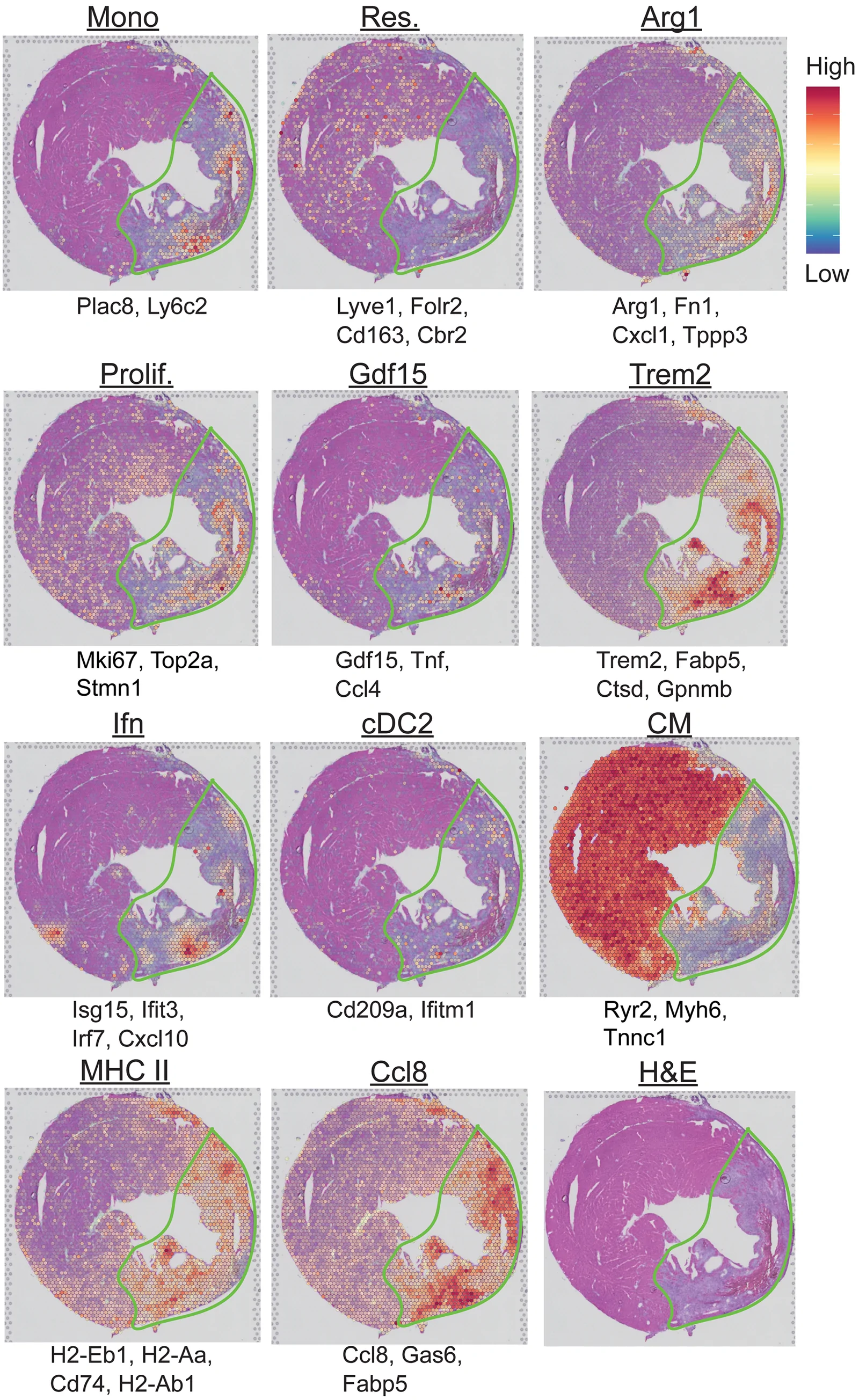
Monocyte-derived populations are spatially distinct. Spatial transcriptomic expression plots of monocyte, macrophage and dependent cell populations, and cardiomyocyte z-scores at day 7 after MI. Images were acquired with a 10X objective.

To assess whether these diverse macrophage populations exist in different regions of the heart, we utilized a published spatial transcriptomic dataset of mouse MI (36). We plotted z-scores of the individual macrophage states onto transverse heart sections at 7 days after MI and observed that individual monocyte, conventional dendritic cells (cDC), and macrophage states were enriched in different regions of the infarct. The infarcted area can be visualized by loss of cardiomyocyte z-score expression. *Arg1^+^*, *Trem2^+^*, *Gdf15^+^*, and *Ccl8^+^* macrophages were enriched near the ischemic core of the infarct, distal to the border zone. Type I IFN–activated macrophages, MHCII^hi^ macrophages, and cDC2s were enriched at the periphery of the infarct nearer to the border zone. Resident macrophages were predominantly found outside of the infarct in the remote zone of the heart, while proliferating macrophages are distributed within the infarct and remote zones (Figure 8). Taken together, these data predict that monocytes differentiate into spatially restricted macrophage subsets and that type I IFN–activated macrophages and *Arg1^+^* macrophages represent distinct intermediate macrophage populations.

### Recruited macrophages begin to acquire transcriptional identity during extravasation.

**Figure 9:**
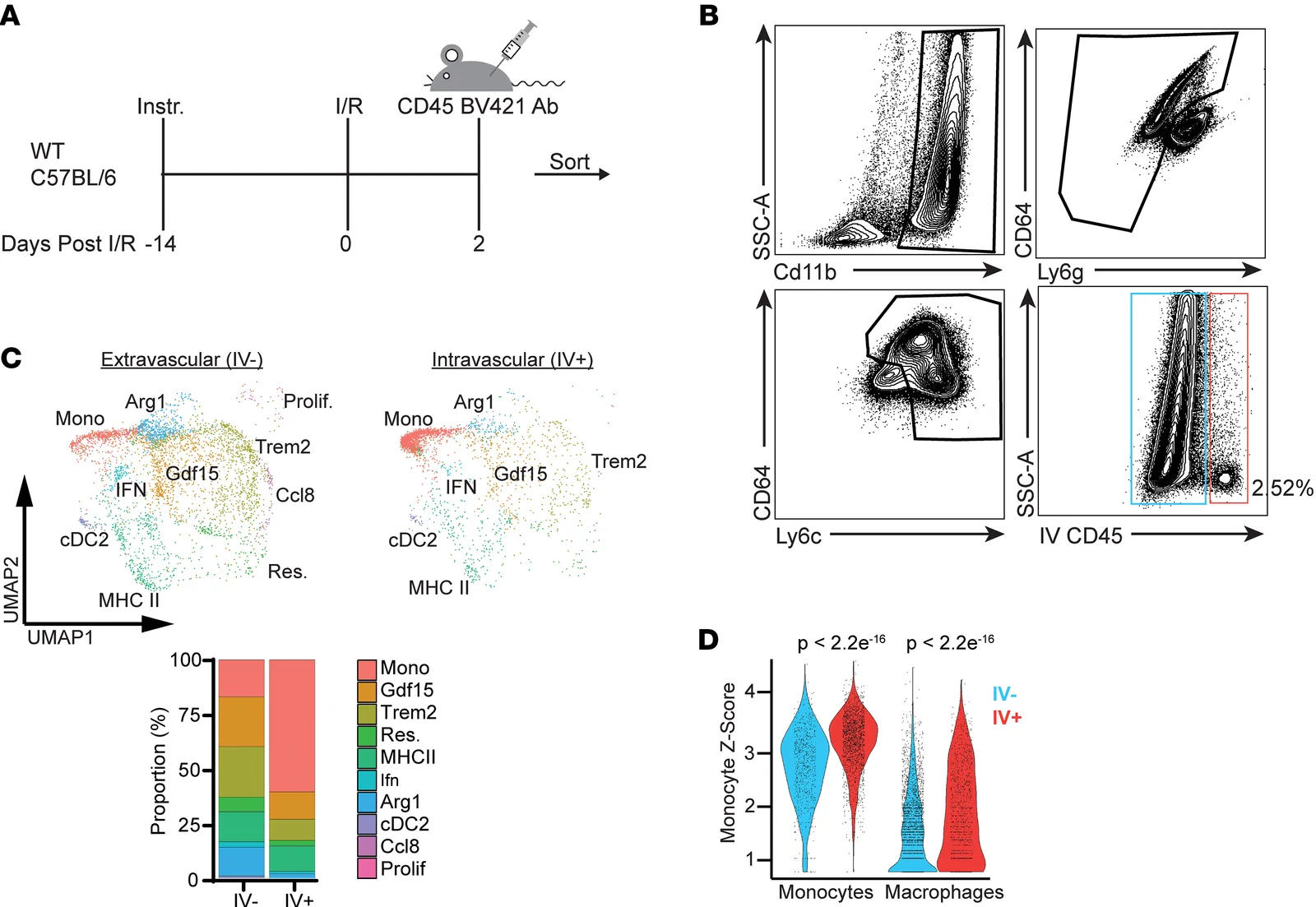
Monocyte fate specifications is initiated prior to myocardial infiltration. (**A**) Experimental strategy for single-cell RNA-seq of intravascular (anti-CD45 BV421+) and extravascular (anti-CD45 BV421–) compartments. Anti-CD45 BV421 was administered via retro-orbital injection 5 minutes prior to collection. (**B**) Representative FACS plots and gating strategy demonstrating anti-CD45 BV421 antibody staining in cardiac monocytes and macrophages. (**C**) Clustered UMAP plots of intravascular (BV421+) and extravascular (BV421–) libraries and stacked bar chart showing distribution of monocyte, macrophage, and dendritic cell populations in each compartment. (**D**) Violin plot of monocyte signature gene expression (expressed as a z-score) in monocytes and macrophages located within the intravascular and extravascular compartments. 2 tailed *t* test with Welch’s correction.

To investigate where fate decisions are initially specified during the process of monocyte recruitment and extravasation into the heart, we performed closed chest ischemia-reperfusion injury on WT mice and harvested animals at 2 days after MI. Mice were administered BV421 conjugated anti-CD45 antibodies retro-orbitally 5 minutes before harvest to label leukocytes in continuity with the circulation (intravascular compartment) (Figure 9A). Upon harvest, hearts were perfused to flush blood cells which had not adhered to the endothelium. Macrophages and monocytes were then sorted by FACS into intravascular (BV421^+^) and extravascular (BV421^–^) libraries for single-cell RNA-seq.

Approximately 2.5% of all macrophages and monocytes collected from the heart were BV421^+^, indicating that these cells had either adhered to the endothelium or were in the process of extravasating from the vasculature into the myocardium (Figure 9B and Supplemental Figure 6). Libraries were sequenced and cell clusters annotated by reference mapping to the *Ccr2^CreERT2^Rosa26^LSL–tdTomato^* monocyte lineage tracing object. As expected, the majority of intravascular cells mapped to the monocyte cluster. Interestingly, we also observed mapping of intravascular cells to each monocyte-derived macrophage populations, suggesting that macrophage fate determination may begin during or prior to monocyte extravasation (Figure 9C). Combined pseudotime analysis using the monocyte lineage tracing and IV anti-CD45 antibody–labeled libraries also demonstrated distribution of intravascular cells throughout all clusters (Supplemental Figure 5, C and D). Consistent with reference mapping, marker genes enriched in each of the monocyte, macrophage, and dendritic cell clusters were expressed within corresponding cells in both the intravascular and extravascular compartments (Supplemental Figure 6). Monocytes and macrophages present within the intravascular compartment displayed transcriptional signatures of monocytes (monocyte z-score), suggesting that they recently differentiated from monocytes (Figure 9D).

**Figure 10:**
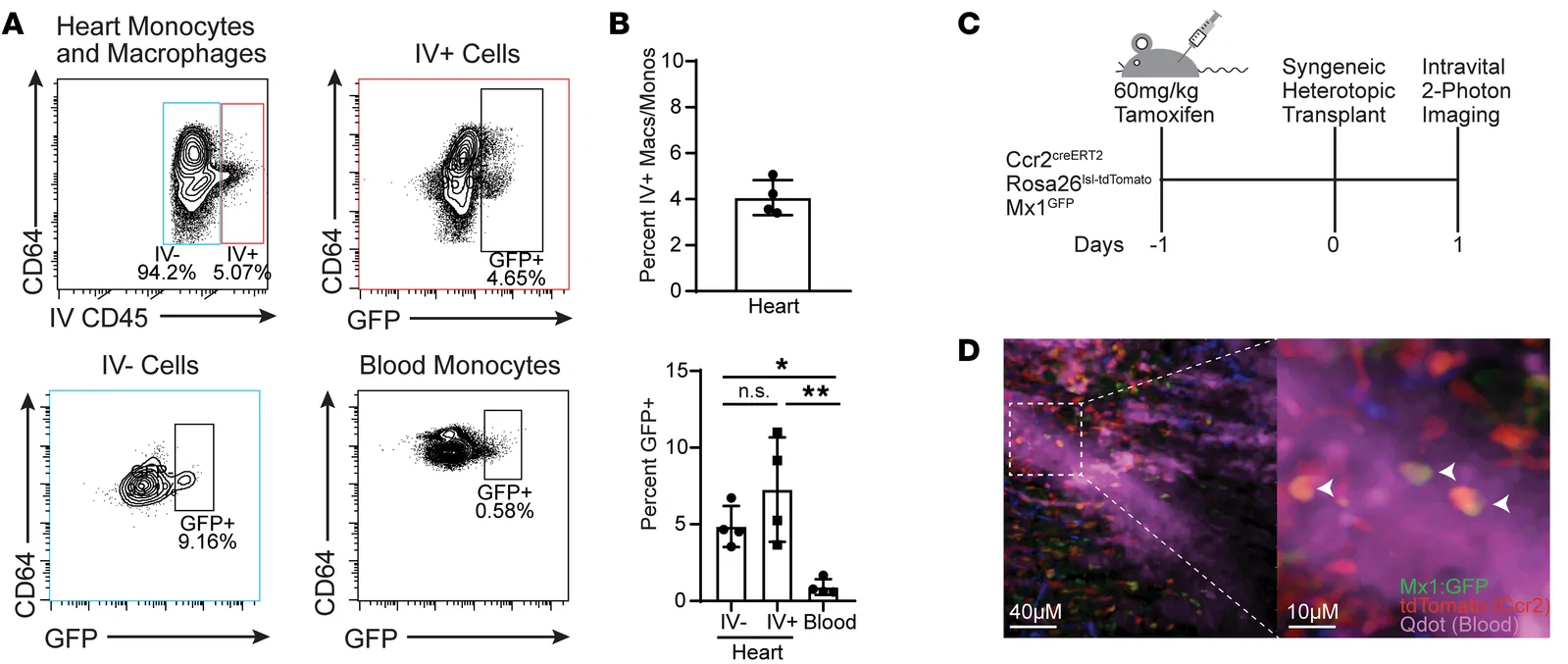
IFN is activated in recruited monocytes during extravasation. (**A** and **B**) Representative FACS plots and quantification of GFP+ monocytes and macrophages within the heart and blood of *Mx1^GFP^* mice after MI. Intravascular and extravascular cardiac monocytes and macrophages expressing GFP is shown. Two tailed *t-*test with Welch’s correction. (**C** and **D**) Experimental strategy for heterotopic heart transplantation and representative images demonstrating *Mx1^GFP^-*positive recruited macrophages (arrowheads) in surface vessels of the WT donor heart. *P < 0.05, **P < 0.01. Scale bars 40 μM (**D**, left); 10 μm (**D**, right).

To corroborate observations from our sequencing data, we performed ischemia reperfusion injury on *Mx1^GFP^* mice (type I IFN reporter) and harvested hearts 2 days after MI. Mice were injected with BV421-conjugated anti-CD45 antibodies 5 minutes before collection. FACS analysis revealed that 4% of monocytes and macrophages within the heart were BV421^+^, indicating that they were localized within the intravascular compartment. GFP-positive cells were evident within the intravascular and extravascular monocyte and macrophage pool. Very few GFP-expressing cells were detected in the peripheral blood. These findings confirm that type I IFN activation within monocytes and macrophages is initiated during extravasation (Figure 10, A and B). To further validate acquisition of fate during extravasation, we performed a syngeneic cervical heterotopic heart transplantation of a WT heart into Ccr2^CreERT2^Rosa25^LSL–tdTomato^*Mx1^GFP^* mice (37). Hearts were subjected to 1 hour of cold ischemia before implantation. After 24 hours, intravital imaging was performed and we observed *Mx1^GFP^* positive macrophages adhered to the vascular endothelium of coronary veins (lumen marked by Q-dots) and extravasated cells within the myocardial interstitial space (Figure 10, C and D, and Supplemental Video 1). Taken together, these results demonstrate that cardiac infiltrating monocytes begin to acquire cell state specific transcriptional identity during extravasation and differentiation into macrophages.

### Type I IFN signaling in monocytes and monocyte-derived macrophages protects from adverse remodeling after MI.

**Figure 11:**
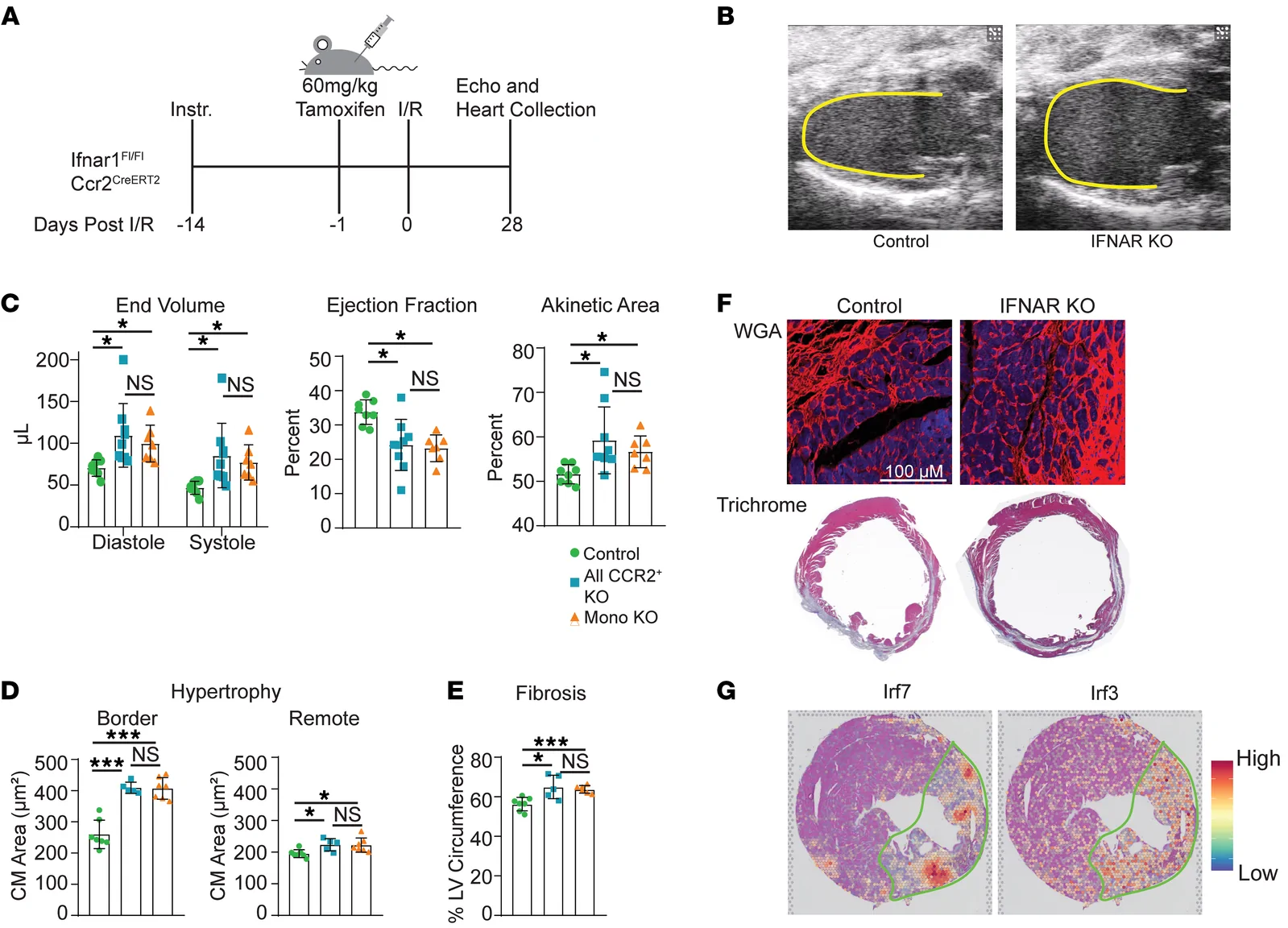
Type 1 IFN signaling in monocytes attenuate post-MI remodeling. (**A**) Experimental strategy for deletion of *Ifnar* in recruited monocytes and their progeny. (**B**) Representative long-axis echocardiographic images at end diastole showing enhanced LV remodeling in *Ccr2^ERT2Cre^Ifnar^Fl^* mice compared to controls. (**C**) Quantification of echocardiography measurements of LV end systolic volume, ejection fraction, and akinetic area 4 weeks after MI. Two tailed t-test with Welch’s correction. (**D**) Measurement of cardiomyocyte size by WGA staining 4 weeks after MI. Two tailed t-test with Welch’s correction. (**E**) Measurement of infarct size assessed by fibrosis as a percentage of LV diameter 4 weeks after MI. Two tailed t-test with Welch’s correction. (**F**) Representative images of WGA staining at the border zone and trichrome staining in control and *Ccr2^ERT2Cre^Ifnar^Fl^* hearts. Scale bar: 100 μM. (**G**) Spatial transcriptomic expression plots of Irf7 and Irf3.

To decipher the functional implications of these diverse macrophage populations, we focused on the IFN-activated macrophage subset given their proximal position within the predicted differentiation trajectory and suggested clinical relevance (38). Utilizing *Ccr2^CreERT2^Ifnar1^Fl^* mice, we selectively deleted *Ifnar1* in recruited monocytes and their progeny using a single dose of tamoxifen, injected IP, referred to as “Mono KO” (Figure 11A) (39). Control and *Ccr2^CreERT2^Ifnar1^Fl^* mice were subjected to MI and echocardiography performed at day 28 after injury. IFNAR1 deletion was validated using flow cytometry of induced peritoneal macrophages (Supplemental Figure 7A). *Ccr2^CreERT2^Ifnar1^Fl^* hearts demonstrated significantly decreased left ventricular ejection fraction, increased end systolic and diastolic left ventricular volumes, and increased akinetic area compared with controls (Figure 11, B and C). Analysis of cardiomyocyte size via wheat-germ agglutinin staining revealed increased cardiomyocyte hypertrophy in the border and remote zones of the left ventricle along with increased fibrosis measured by trichrome staining. We observed consistent findings in *Ccr2^CreERT2^Ifnar1^Fl^* mice subjected to continuous tamoxifen treatment, which will delete *Ifnar1* in resident CCR2^+^ macrophages, monocytes, and their progeny, referred to as “All CCR2^+^ KO”. (Figure 11, D–F). These data are consistent with the conclusions that type I IFN primarily functions within monocytes and their progeny recruited to the heart early after injury and that type I IFN signaling within infiltrating monocytes and their progeny confers a protective effect on MI remodeling. Intriguingly, these findings are in contrast to studies demonstrating protective effects in global *Ifnar1^–/–^* mice, suggesting that type I IFN signaling has cell type–specific effects following MI (38). We next utilized spatial transcriptomics to delineate different type I IFN signaling pathways. We observed that *Irf3* expression was observed throughout the infarct, while *Irf7* expression was enriched in the border zone Figure 11G). IRF3 was reported to regulate IFN activation in response to cytoplasmic DNA sensing (38, 40, 41). In contrast, IRF7 functions downstream TLRs and is amplified in response to type I IFN signaling. The expression of IRF7 is consistent with the most robust IFN signal in the border zone, which is the location of type I IFN–activated macrophages.

### Type I IFN signaling is essential for the differentiation of MHCII^hi^ macrophages.

**Figure 12:**
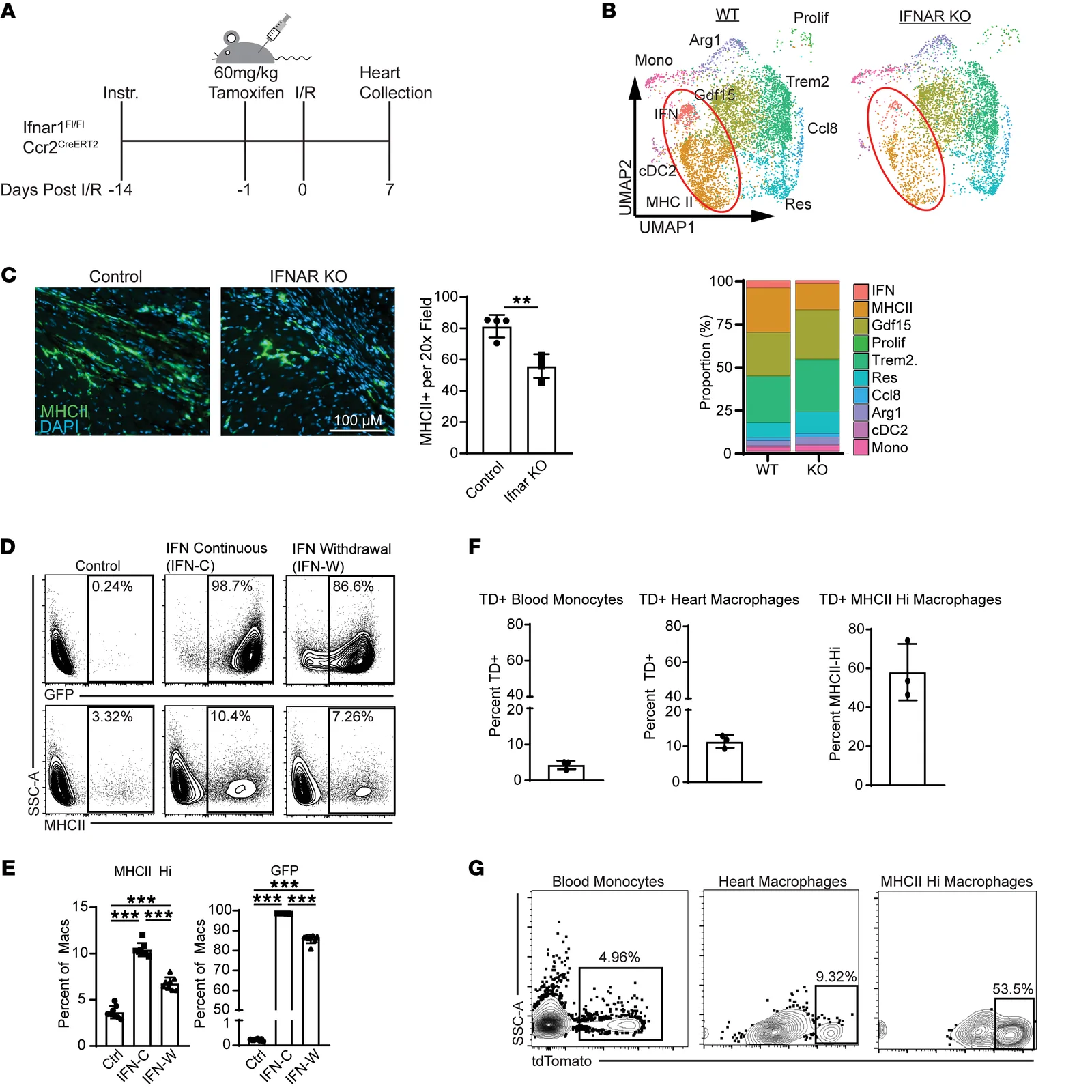
Type 1 IFN–activated macrophages give rise to MHCII^hi^ macrophages. (**A**) Experimental strategy for deletion of *Ifnar* in recruited monocytes and their progeny. (**B**) UMAP projection and stacked bar chart comparing distribution of cells in control and *Ccr2^ERT2Cre^Ifnar^Fl^* hearts. (**C**) Representative images and bar chart quantifying MHCII+ macrophages per 20 × field in the infarct of control and *Ccr2^ERT2Cre^Ifnar^Fl^* hearts. 2 tailed *t* test with Welch’s correction. Scale bar: 100 μM. (**D** and **E**) FACS plots of elicited peritoneal macrophages isolated from Mx1^GFP^ mice stimulated with either PBS or IFN-β showing GFP and MHC-II expression (**D**). Quantification of the percent GFP+ and MHCII+ macrophages (**E**). 2 tailed *t* test with Welch’s correction. (**F** and **G**) Quantification and representative FACs plots demonstrating *Mx1^ERT2Cre^Rosa26^LSL–tdTomato^* labeling in circulating monocytes, macrophages in the heart, and percent of tdTomato^+^ macrophages which were MHCII^hi^.

We next sought to determine the effect of monocyte *Ifnar1* deletion on the diversity of recruited monocytes and macrophages. We performed ischemia reperfusion injury on control and *Ccr2^CreERT2^Ifnar1^Fl^* mice given a single IP injection of tamoxifen 1 day prior to injury. Hearts were collected 7 days after injury and processed for single-cell RNA-seq (Figure 12A). Libraries were sequenced and clusters annotated by reference mapping to the *Ccr2^CreERT2^Rosa26^LSL–tdTomato^* object. As expected, the proportion of IFN-activated macrophages was reduced. Interestingly, the proportion of MHCII^hi^ macrophages was also dramatically reduced in *Ccr2^CreERT2^Ifnar1^Fl^* mice compared with litter mate controls (Figure 12B and Supplemental Figure 7B). Combined pseudotime analysis using monocyte lineage tracing, IV labeling, and *Ccr2^CreERT2^Ifnar1^Fl^* libraries demonstrated a reduction in terminal MHCII^hi^ macrophages (Supplemental Figure 7C). Immunostaining confirmed that MHCII^hi^ macrophages were significantly reduced in *Ccr2^CreERT2^Ifnar1^Fl^* hearts compared with controls at 28 days after injury (Figure 12C).

To assess whether type I IFN signaling to monocytes directly influences MHCII^hi^ macrophage differentiation, we utilized thioglycolate-induced peritoneal macrophages (pMacs) from *Mx1^GFP^* mice. pMacs were cultured in complete media with 25 nM IFN-β for 3 days followed by 4 days without IFN-β (IFN Withdrawal) or for 7 days with IFN-β (IFN Continuous). After 7 days of culture, pMacs were collected and flow cytometry was performed to identify percentages of GFP^+^ and MHCII^hi^ macrophages. Continuous treatment with IFN-β resulted in GFP expression in 98.7% of pMacs compared with less than 0.25% of controls. Continuous treatment also significantly increased the proportion of MHCII^hi^ pMacs from 3.7% to 10.4%. Withdrawal of IFN-β reduced the percentage of MHC^II^ high macrophages showing a reversible effect (Figure 12, D and E). These results indicate that type I IFN signaling directly regulates the acquisition of the MHCII^hi^ macrophage fate.

We additionally generated an *Mx1^CreERT2^Rosa26^LSL–tdTomato^* reporter line to evaluate contribution of IFN-activated macrophages to other macrophage populations. Using an angiotensin II/phenylephrine infusion model of cardiac injury in conjunction with flow cytometry, we found that less than 5% of circulating monocytes were tdTomato^+^ and that 10% of all macrophages in the heart were tdTomato^+^. Importantly, 60% of tdTomato^+^ macrophages were MHCII^hi^, indicating that IFN-activated macrophages give rise to MHCII^hi^ macrophages and represent a major contributor to this population (Figure 12, F and G).

### Mechanisms by which type I IFN signaling in macrophages confers protection.

To investigate mechanisms by which IFN-activated macrophages are protective in myocardial infarction, we (a) assessed the role of MHC-II expression (regulated by type I IFN signaling) in monocyte-derived macrophages and (b) explored cell types that may interact with type I IFN–activated macrophages. We first investigated the functional role of MHCII in monocyte-derived macrophages following myocardial injury. Utilizing *Ccr2^CreERT2^MHCII^Fl^* mice, we selectively deleted *MHCII* in recruited monocytes and their progeny using a single dose of tamoxifen, injected IP (Supplemental Figure 8A) (42). Control and *Ccr2^CreERT2^MHCII^Fl^* mice were subjected to reperfused MI and echocardiography performed at day 28 after injury. *Ccr2^CreERT2^MHCII^Flx^* hearts demonstrated significantly decreased left ventricular ejection fraction, increased end systolic and diastolic left ventricular volumes, and increased akinetic area compared with controls (Supplemental Figure 8, B and C). Analysis of cardiomyocyte size via wheat-germ agglutinin staining revealed increased cardiomyocyte hypertrophy in the border and remote zones of the left ventricle; however, no change in fibrosis was observed (Supplemental Figure 8, D–F). These results demonstrate that MHC-II expression in monocyte-derived macrophages is protective in myocardial infarction and are consistent with prior reports indicating that antigen-specific CD4^+^ T cells may be protective in the heart (43).

**Figure 13:**
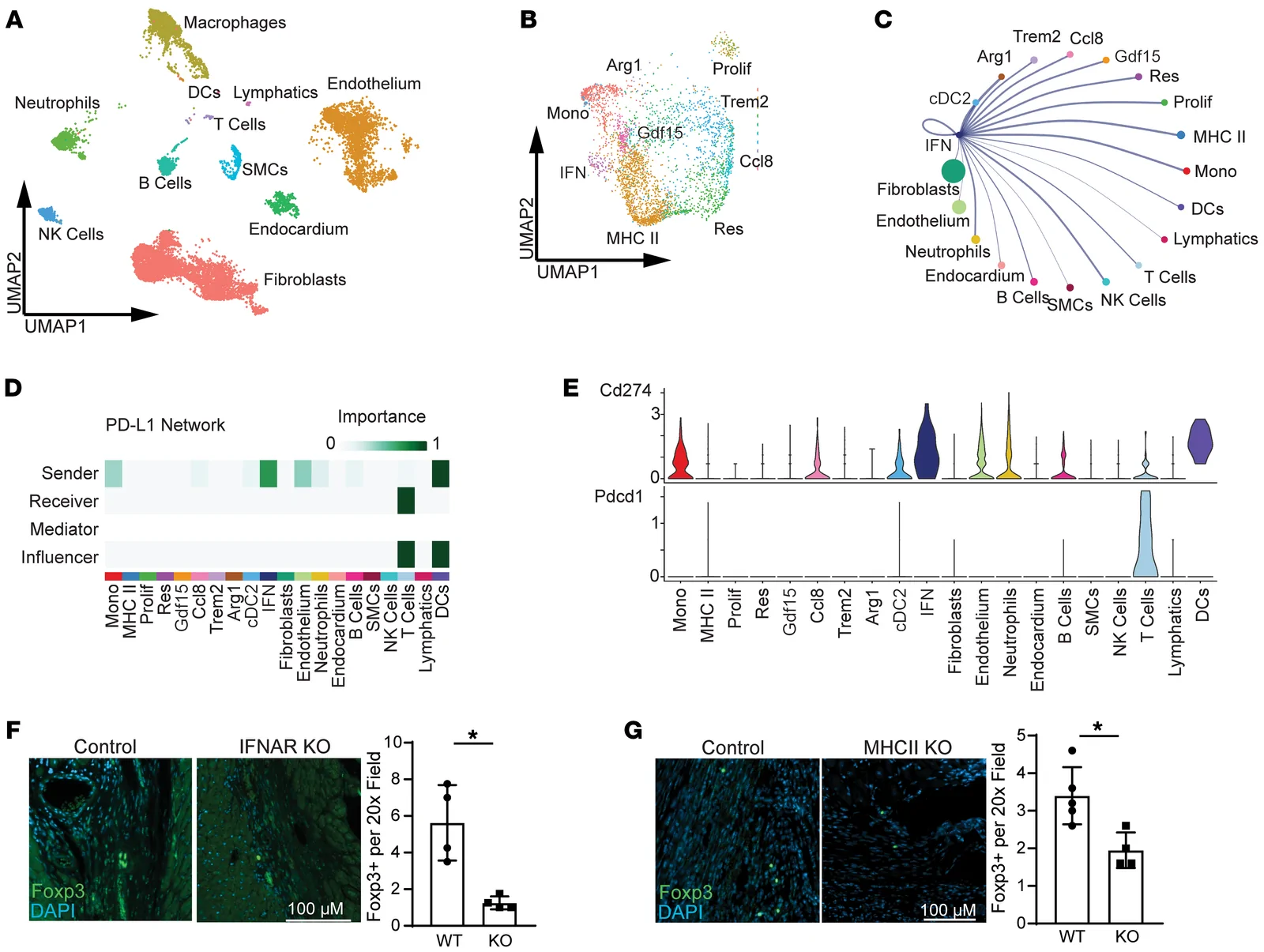
Type 1 IFN–activated macrophages promote Foxp3+ Treg development. (**A**) UMAP projection of single-cell data of all cell types obtained from hearts at D8 after MI. (**B**) UMAP projection of macrophages from experiment in panel A reference mapped to the monocyte lineage tracing dataset. (**C**) CellChat diagram demonstrating signaling from IFN-activated macrophages. (**D**) Heatmap of the PD-L1 signaling network. (**E**) Violin plot of Pd-L1 (Cd274) and Pd1 (Pdcd1). (**F)** Representative images and bar graph quantifying Foxp3+ cells per 20 × field in the infarct in control and *Ccr2^ERT2Cre^Ifnar^Fl^* hearts. (**G**) Representative images and bar chart quantifying Foxp3+ cells per 20X field in the infarct in control and *Ccr2^ERT2Cre^Ab1^Fl^* hearts. *P < 0.05, **P < 0.01, ***P < 0.001 Two tailed t-test with Welch’s correction. Scale bars: 100 μm.

We then utilized a single-cell RNA-seq dataset encompassing stromal cell types in the heart at 8 days after ischemia-reperfusion injury (Figure 13A). We referenced mapped macrophages from this dataset to derive annotations and performed cell-cell communication analysis using CellChat (Figure 13B) (44). We observed predicted communication between IFN-activated macrophage and multiple other cell types within the heart (Figure 13C). Recent studies have demonstrated the importance of regulatory T cells (Tregs) in dampening inflammation and remodeling in myocardial infarction (45). This led us to evaluate the PD-L1 signaling pathway, which is responsible for promoting of Treg specification. We identified that IFN-activated macrophages, along with dendritic cells, are the primary producers of PD-L1 among macrophage populations (Figure 13, D and E). To evaluate the impact of *Ifnar1* deletion in monocyte-derived macrophages on Treg abundance, we performed immunostaining for FOXP3 (Treg marker) and observed that Ifnar1 deletion in monocyte-derived macrophages resulted in significantly reduced numbers of Tregs in the infarct at 28 days after injury (Figure 13F) (46). Similarly, MHC-II deletion results in reduced numbers of Tregs, consistent with evidence in cancer that MHC-II^hi^ macrophages can induce Treg development (Figure 13G) (47). To elucidate the source of type I IFN in ischemic injury, we utilized a publicly available human single nuclei RNA-seq dataset (48) This dataset demonstrated elevated levels of type I IFN ligands, (particularly *IFNB1*) across cardiac cell types in ischemic samples, indicating ubiquitous induction of type I IFN signaling in acute myocardial infarction (Supplemental Figure 9). These results indicate that IFN activated macrophages directly contribute to the development of Tregs through PD-L1 expression, providing a plausible explanation for the observed protective effects on inflammation and remodeling.

## Discussion

This study provides insights into the spatiotemporal dynamics of monocyte recruitment and differentiation following MI. Monocytes are recruited into the myocardium within the first 5–7 days after MI, and their progeny persisted through at least 28 days after MI. Single-cell RNA-seq combined with genetic lineage tracing provided an unprecedented opportunity to precisely define the fates of monocytes that infiltrate the infarcted heart. This technique enabled the identification of monocyte, macrophage, and cDC2 populations that reside within the infarcted heart and demonstrated a coordinated and dynamic shift in macrophage phenotype over time. Furthermore, we demonstrated that these diverse macrophage subsets are situated in distinct locations within the heart, begin to acquire their transcriptional identity during extravasation, and that type I IFN–activated macrophages and *Arg1*^+^ macrophages are predicted as intermediate populations that give rise to more terminal macrophage states. Within this framework, type I IFN signaling in infiltrating monocytes and derived macrophages promotes the differentiation of MHC-II^hi^ macrophages and confers protection following MI. These findings uncover the complexity of monocyte fate decisions and highlight the therapeutic potential for targeting monocyte differentiation.

In recent years, numerous publications have demonstrated the heterogeneity of macrophages in the heart and other organs using single-cell RNA-seq. Of note is the dramatically distinct transcriptional profiles taken on by recruited macrophages after tissue injury. However, little is known about when and how these distinct phenotypes are derived. To evaluate spatial localization of differentiation, we leveraged a published spatial transcriptomics dataset of mouse MI that demonstrated distinct regions of differentiation relative to the infarcted area (36). Evaluating pseudotime identified intermediate IFN-activated and *Arg1*^+^ macrophage populations and revealed that these populations are concentrated near the border zone and ischemic core of the infarct, respectively. This finding is consistent with previous studies demonstrating clustering of the type I IFN response in the border zone (41). These locations correspond to terminal populations also found in these regions — MHCII^hi^ in the border zone and *Trem2*^+^ in the core — suggesting that localization within the infarct may influence the trajectory of recruited macrophages.

With respect to timing of fate acquisition, we observed that the majority of extravasating cells retained a monocyte expression profile. Retained expression of monocyte markers within intravascular macrophages is also consistent with the hypothesis that monocytes begin to express markers of specific macrophage populations during extravasation. Activation of the IFN reporter *MX1* in extravasating cells but not in the bone marrow further supports this hypothesis. A recent study using permanent ligation myocardial infarction in mice demonstrated IFN stimulated gene (ISG) expression in monocyte and neutrophil progenitors in the bone marrow as well as expression of ISGs in human peripheral blood neutrophils (49). While our findings do not identify activation of IFN signaling in peripheral blood monocytes, they also do not rule out that activation within the bone marrow is possible in different injury models or using different detection methods. Future studies are needed to determine when during the extravasation process macrophage fate specification begins.

This work lays the foundation for constructing monocyte differentiation trajectories. By analyzing single cell expression profiles at multiple time points after reperfused MI, we have demonstrated that recruited monocyte-derived macrophages are retained in tissue and alter their expression profile over time. The data presented here along with that in another recent publication (28), indicate that recruited monocytes differentiate into at least 2 intermediate states: *Arg1^+^* macrophages and type I IFN–activated macrophages. We previously demonstrated through direct lineage tracing that *Arg1^+^* macrophages give rise to multiple additional macrophage subsets including *Gdf15^+^*, *Trem2^+^*, MHCII^hi^, and resident-like macrophages (28). Here, we demonstrate that IFN-activated macrophages give rise to MHCII^hi^ macrophages. This concept is supported by a dramatic decrease in MHCII^hi^ macrophages when *Ifnar1* is deleted in monocytes and derived macrophages, the observation that IFN-β treatment of elicited peritoneal macrophages in vitro is sufficient to induce expression of MHCII, and direct lineage tracing of type I IFN–activated macrophages demonstrating labeling of the majority of MHCII^hi^ macrophages. Additional studies and analysis at additional time points are needed to directly trace the fates of other monocyte-derived subsets to definitively understand whether they represent an intermediate or terminally differentiated state and understand their plasticity and differentiation mechanisms. The mechanisms underlying regulation of other monocyte and macrophage fate transitions are indeed the subject of several ongoing studies. Future experiments utilizing unbiased lineage tracing techniques will further clarify trajectories of monocyte differentiation.

Previous studies have demonstrated that type I IFN signaling may have detrimental effects in the heart. MI induces a feed-forward type I IFN inflammatory response triggered by sensing of cytoplasmic DNA and activation of the cGAS-STING pathway in cardiomyocytes. Global deletion of STING, IRF3, and IFNAR in mice display reduced incidence of cardiac rupture, increased survival, and impaired wound healing after permanent left coronary artery ligation (38, 41). Similarly, pressure overload by transverse aortic constriction in mice also induces activation of type I IFN, reduced contractile function, and impaired sarcomeric turnover, phenotypes rescued by knockout of *Isg15* (50). Conversely, our results demonstrate a cardioprotective effect of type I IFN signaling in monocytes and monocyte-derived macrophages, as evidenced by decreased left ventricular systolic function and accelerated cardiac remodeling after reperfused MI. Prior studies have also suggested that type I IFN activation initiates within cardiomyocytes at the border zone (41). Our data suggest that initiation of signaling is distinct within different regions of the infarct based on spatially distinct expression of *Irf3*, which regulates IFN signaling through sensing of cytoplasmic DNA, and *Irf7*, which functions downstream of TLRs in response to type I IFN signaling. This indicates that, while the cascade likely begins with cardiomyocytes at the border zone and this initial signaling is detrimental to the heart, later activation in macrophages is beneficial to preserving cardiac function. The ubiquitous nature of different cell types capable of responding to type I IFN signaling and their varied responses warrants further study. Additionally, we demonstrate that MHCII^hi^ macrophages are protective in ischemic cardiac injury. Future studies will further investigate the relationship between IFN-activated and MHCII^hi^ macrophages and the mechanisms by which they confer cardioprotection.

Beginning with cancer treatments, modulation of T cell activation and fate has become of great clinical interest. Immune checkpoint blockade has been revolutionary in treating cancer by allowing T cells to engage with tumor cells (51). Further, chimeric antigen receptor (CAR) T cells initially approved for treatment of cancer are being investigated for numerous other treatments, including for treatment of cardiac injury (52, 53). These highlight the potential for modulating T cells as therapeutic agents. We demonstrate that type-I IFN activated macrophages express the highest levels of PD-L1 among macrophage populations and that they directly effect the generation of regulatory T cells, which have been demonstrated to prevent remodeling, highlighting the possibility of generating T cell–based therapies for treating cardiac injury (43).

This study is not without limitations. Computational analysis only predicts differentiation trajectories and relationships between each monocyte, macrophage, and dendritic cell population. While targeted lineage tracing has demonstrated apparent trajectories within these heterogenous macrophage populations, unbiased genetic fate mapping is ultimately required to refine and validate our findings. The signaling pathways by which macrophage fates are specified within the vascular compartment remain obscure and will undoubtedly be the topic of future studies. The precise mechanism by which IFN signaling orchestrates the differentiation of MHCII^hi^ macrophages after MI is of great interest. The mechanisms by which IFN-activated macrophages interact with and modulate regulatory T cell function will be the focus of future studies and together could lead to novel therapeutic insights.

In conclusion, we demonstrate that monocyte-derived macrophages are recruited to the heart within the first week following MI and differentiate into evolutionarily conserved, spatially restricted, and transcriptionally diverse array of distinct macrophage and dendritic cell–like states. We determined that monocyte fate specification begins prior to entry into tissue and identified a potentially novel role of type I IFN signaling in recruited monocytes and macrophages that governs the differentiation of MHCII^hi^ macrophages, promotes regulatory T cell development, preserves left ventricular systolic function, and prevents cardiac adverse remodeling. Together, our findings provide what we believe to be novel and clinically relevant insights that suggest monocyte fate specification and modulation may serve as a target of immunomodulatory therapy.

## Methods

### Sex as a biological variable.

For all experiments involving mouse models, equal numbers of male and female mice were included to ensure results could be generalized across sexes. However, data from both sexes were combined for the final analyses without evaluating sex-dependent differences and were not interpreted relative to sex.

### Animal models.

Mus musculus were housed in ultraclean rodent barrier facility with veterinarians from the Washington University Division of Comparative Medicine on call or on site for animal care. All procedures were performed in accordance with IUCAC protocols. Strains used were *Cx3cr1^ERT2Cre^* (54) (JAX #020940), *Rosa26^lox–stop–lox–tdTomato^* (24) (JAX #007914), *CCR2^ERT2Cre^* (55), *Mx1^GFP^* (56) (JAX #033219)*, MhcII^Fl^* (42) (JAX #037709), and *Ifnar1^Fl^* (39) (JAX #028256) on the C57BL/6 background. *Mx1^ERT2Cre^* were generated in collaboration with The Jackson Laboratories using CRISPR/Cas9 to insert ERT2Cre into the translation start site of the *Mx1* gene as was previously targeted in generation of the *Mx1^GFP^* strain. Experiments were performed on mice 6-12 weeks of age. Equal numbers of male and female mice were used for experiments. For Cre recombination in all monocytes and macrophages, *Ccr2^CreERT2^ Ifnar1^fl/fl^* mice were given tamoxifen food pellets (Envigo TD.130857) for the entirety of the experiment. For Cre recombination in other lines, mice were given IP injection of 60 mg/kg of tamoxifen (Millipore Sigma T5648) at the indicated frequency and timing.

### Closed-chest ischemia reperfusion injury.

Mice were anesthetized, intubated, and mechanically ventilated. The heart was exposed, and a suture placed around the proximal left coronary artery. The suture was threaded through a 1 mm piece of polyethylene tubing to serve as the arterial occlude. Each end of the suture was exteriorized through the thorax. The skin was closed, and mice were given a 2-week recovery period prior to induction of ischemia. After 2 weeks, the animals were anesthetized and placed on an Indus Mouse Surgical Monitor system to accurately record ECG during ischemia. Ischemia was induced after anesthetizing the animals. Tension was exerted on suture ends until ST-segment elevation was seen via ECG. Following 90 minutes of ischemia time, tension was released, and the skin was then closed.

### Heterotopic heart transplantation and two-photon intravital imaging.

For *Mx1^GFP^* intravital two-photon imaging following syngeneic heart transplant, established protocols were used (57–59). Cardiac grafts were collected from donor wild type mice and transplanted into the cervical position of recipient *Mx1^GFP^* mice after 1 h of cold (4°C) ischemia. The donor ascending aorta and pulmonary artery were connected to the recipient right common carotid artery and right external jugular vein. At 24 h after syngeneic heart transplantation, mice were anesthetized with ketamine (80 mg/kg) and xylazine (10 mg/kg), intubated, and connected to a mouse ventilator. Then, 655nm nontargeted Qdots (Thermo Fisher Scientific) were injected through the intravenous route into recipient mice before imaging. The heart was fixed to an imaging chamber and imaged for 15 min. Time-lapse imaging was performed with a custom-built two-photon microscope running ImageWarp version 2.1 acquisition software (A&B Software). Imaging is terminal and mice were euthanized.

### Echocardiography.

Mice were sedated with Avertin (0.005 ml/g) and 2D and M-mode images were obtained in the long and short axis views 4 weeks after MI using the VisualSonics770 Echocardiography System. Left diastolic volume, left systolic volume, akinetic area, and total LV area were measured using edge detection and tracking software (VivoLab). Ejection fraction was calculated as the (left diastolic volume – left systolic volume) / left diastolic volume. Akinetic percentage was calculated as the akinetic area / total LV area.

### Histology.

4 weeks after MI, mice were euthanized, and hearts perfused with PBS. Hearts were fixed overnight with 4% PFA in PBS at 4°C, cut into thirds on the transverse plane, placed into histology cassettes for paraffin embedding, and dehydrated in 70% ethanol in water. Hearts were paraffin embedded and cut into 4 μm sections and mounted on positively charged slides. Tissues were stained by Gomori’s Trichrome staining (Richard-Allan Scientific 87020) and WGA staining (Vector Laboratories RL-1022). Trichrome staining was imaged using a Meyer PathScan Enabler IV. Measurement of infarct length and LV length was performed in FIJI, with data quantification normalizing the length of the infarct to the length of the LV (60). WGA staining was imaged using the 20x objective of a Zeiss Axioscan7 confocal microscope. 3 20x fields of the border zone of the infarct were randomly selected per mouse. 20 cross sectional (not longitudinal) cardiomyocytes per 20x field were selected at random. Measurement of cross-sectional cardiomyocyte area was performed in FIJI. Data is displayed as the average cardiomyocyte cross sectional area per mouse.

### Immunofluorescence.

Mice were euthanized, and hearts perfused with PBS. Hearts were cut midway on the transverse plane and fixed overnight with 4% PFA in PBS at 4°C. They were then dehydrated in 30% sucrose in PBS overnight at 4°C. Hearts were embedded in O.C.T. (Sakura 4583) and frozen at –80°C for 30 minutes. Hearts were then sectioned at 15 μm using a Leica Cryostat, mounted on positively charged slides, and stored at –80°C. For staining, the following steps were performed at room temperature and protected from flight. Slides were brought to room temperature for 5 minutes and washed in PBS for 5 minutes. Sections were then permeabilized in 0.25% Triton X in PBS for 5 minutes. Sections were then blocked in 5% BSA in PBS for 1 hour. Sections were then stained with primary antibody diluted in 1% BSA in PBS for 1 hour with rat anti CD68 (BioLegend 137002 1:400 dilution), rabbit anti Lyve1 (Abcam ab14917 1:200 dilution). Sections were then washed 3 times 5 minutes each with PBS-Tween. After washing, the appropriate secondary antibodies were added diluted in 1% BSA in PBS for 1 hour at a 1:1000 dilution (Goat anti Rat 488 Life Technologies a11006, Donkey anti Rabbit 647 Abcam ab150075,). Slides were again washed 3 times 5 minutes each with PBS-T, and then mounted with DAPI mounting media (Millipore Sigma F6057) and cover slipped. Slides were stored at 4°C protected from light and imaged within 1 week.

Slides were imaged using the 20x objective of a Zeiss Axioscan7 microscope. Visualization of tdTomato was from the endogenous signal. For quantification, 3-5 20x fields were randomly selected per mouse of either the remote zone or infarct zone as indicated. Quantification of cell numbers were performed in FIJI or Zeiss Zen, and data is displayed as the average of each 20x field per mouse.

### Flow cytometry.

Hearts were perfused with PBS, weighed, minced, and digested for 45 minutes at 37°C in DMEM (Gibco 11965-084) containing 4500 U/ml Collagenase IV (Millipore Sigma C5138), 2400 U/ml Hyaluronidase I (Millipore Sigma H3506), and 6000 U/ml DNAse I (Millipore Sigma D4527). Enzymes were deactivated with HBSS containing 2% FBS and 0.2% BSA and filtered through 40-micron strainers. Cells were incubated with ACK lysis buffer (Gibco A10492-01) for 5 minutes at room temperature. Cells were washed with DMEM and resuspended in 100 μl of FACS buffer (PBS containing 2% FBS and 2mM EDTA).

Approximately 100 μl of blood was collected by cheek bleed into a tube containing 20 μl of EDTA (Corning 46-034-CI). Cells were incubated with ACK lysis buffer for 15 minutes at room temperature, washed, and ACK lysed a second time. Cells were then washed with DMEM and resuspended in 100 μl of FACS buffer.

Peritoneal macrophages were induced by I.P. injection of 1ml of thioglycollate medium. Four days after injection, mice were euthanized and 10ml of sterile PBS was injected into the peritoneal cavity and then withdrawn. Cell suspension was treated with ACK lysis buffer, washed with DMEM, and resuspended in 100 μl of FACS buffer.

Cells were incubated in a 1:200 dilution of fluorescence conjugated monoclonal antibodies for 30 minutes at 4°C. Samples were washed in FACS buffer and resuspended in a final volume of 250 μl FACS buffer. Flow cytometry and sorting was performed using a BD FACS melody.

For sorting of monocytes and macrophages from *Cx3cr1^CreERT2^Rosa26^LSL–tdTomato^* for single cell RNA sequencing, antibodies used were: CD45 PerCP/Cy5.5 (BioLegend 103132), LY6G APC/Cy7 (BioLegend 127623), Ly6C APC (BioLegend 128016), CD64 PE/Cy7 (BioLegend 139313), and CD11B BV421 (BioLegend 101235).

For sorting of monocytes and macrophages from *Ccr2^CreERT2^Rosa26^LSL–tdTomato^* for single cell RNA sequencing, antibodies used were: CD45 PerCP/Cy5.5 (BioLegend 103132), LY6G APC/Cy7 (BioLegend 127623), LY6C FITC (BioLegend 128005), and CD64 APC (BioLegend 164409), CD11B PE/Cy7 (BioLegend 101215), CD3 BV510 (BioLegend 100233), CD19 BV510 (BioLegend 115545) and MHCII BV421 (BioLegend 107631).

For flow cytometry of blood from *Ccr2^CreERT2^Rosa26^LSL–tdTomato^*, antibodies used were: CD45 PerCP/Cy5.5 (BioLegend 103132), LY6G BV421 (BioLegend 127627), LY6C PE/Cy7 (BioLegend 128017), CD115 APC (BioLegend 135509), CD3 FITC (BioLegend 152303), and CD19 BV510 (BioLegend 115545).

For flow cytometry of bone marrow from *Ccr2^CreERT2^Rosa26^LSL–tdTomato^*, antibodies used were: SCA1 FITC (BioLegend 108105), C-KIT PerCP/Cy5.5 (BioLegend 135133), IL7RA PE/Cy7 (BioLegend 158209), CD34 APC (BioLegend 119309), CD16/CD32 APC/Cy7 (BioLegend 156611), and Lineage Cocktail BV421 (BioLegend 133311).

### Single cell RNA sequencing analysis.

Monocytes and macrophages were sorted after I/R as indicated. Single cell RNA sequencing libraries were generated using the 10X Genomics 5’v1 platform. Libraries were sequenced using a NovaSeq 6000 at the McDonnel Genome Institute (MGI). For *Cx3cr1^CreERT2^Rosa26^LSL–tdTomato^* mice, cells were separated into tomato- and tomato+ libraries. Sequencing reads were aligned to the GRCm38 transcriptome using CellRanger (v3.1.0) from 10X Genomics. Custom

Downstream data processing was performed in the R package Seurat (v4) (61). SCtransform was used to scale and normalize the data for RNA counts. Quality control filters of genes/cell>200, mitochondrial reads/cell<10%, read counts/cell>5000, and read counts/cell<40000 were applied. PCA was used for dimensionality reduction, data was clustered at multiple resolutions, and UMAP projections were generated for data visualization. Harmony integration was applied to account for batch effect from different time points (62). Contaminating populations of neutrophils and non-myeloid cells were excluded from analysis. Differential gene expression of upregulated genes in each subpopulation was generated in Seurat using FindAllMarkers using the Wilcoxan Rank Sum Test with a minimum fraction (min.pct) of 0.1 and a logFC threshold of 0.1. Gaussian kernel density estimation plots were generated using the Python package Scanpy (v1.8.1) (63).

### RNA velocity analysis.

The ScVelo Python package was used to generate loom files with spliced and unspliced read counts for each library (33). Velocyto.R package for R was then utilized to perform RNA velocity analysis using the TSNE generated by Palantir (34). RunVelocity command was performed using default parameters of spliced.average = 0.2 and unspliced.average = 0.05. Velocity is plotted on the TSNE using the show.velocity.on.embedding.cor command using a neighborhood size of 200 cells and square-root scaling of velocity.

### Palantir trajectory analysis.

The Palantir Python package was used for trajectory analysis (v1.0.0 to v1.1) (35). Prior to inputting data to Palantir, cell cycle regression was performed using Seurat and proliferating cells were excluded from pseudotime analysis. Monocytes were used as the early and starting cell type for Palantir. Highly variable gene analysis was not used. The eigenvectors used was determined by the eigengap. The number of waypoints used was 500. The option to use the early cell as the start cell was used. The output pseudotime/entropy values, terminal state probabilities, and TSNE coordinates for each cell were used to generate plots in R.

### Spatial transcriptomics.

Spatial transcriptomics data of macrophage sub-population localization was generated using a published data set of mouse MI 7 days after injury (GSE176092) (36). Spatial data was analyzed using Seurat. The macrophage population gene signature was plotted by calculating the Z-score of the genes as listed and using the function “SpatialFeaturePlot” using the Z-score as a feature.

### Statistics.

For analysis of echocardiography, histology, and immunofluorescence, 2 tailed *t* tests with Welch’s correction were performed in GraphPad Prism. At least 3 replicates were used for experiments. For percentage of tdTomato-positive cells by FACS and immunofluorescence, a Brown-Forsythe 1-way ANOVA was performed in Prism. *P* < 0.05 was a priori considered statistically significant. Data was plotted using Prism software.

### Study approval.

Animal studies were performed in compliance with guidelines set forth by the National Institutes of Health Office of Laboratory Animal Welfare and approved by the Washington University institutional animal care and use committee.

### Data availability.

Single Cell RNA sequencing data are available on the Gene Expression Omnibus (GSE252086). The Supporting Data Values file contains data used in generating graphs and performing statistics. R and Python scripts are available on GitHub (https://github.com/alkoenig/Cardiac_Monocyte_Fate/commit/2e82700, Commit ID:2e82700).

## Author contributions

ALK: conceptualization, data curation, formal analysis, funding acquisition, investigation, methodology, supervision, validation, visualization, writing – original draft, writing – review & editing. FFK: conceptualization, data curation, formal analysis, investigation. JMA: data curation, formal analysis, investigation. SY: data curation, formal analysis, investigation. CJW: data curation, investigation. JMN: data curation, investigation. AK: data curation, investigation. WL: data curation, investigation. GBS: data curation, formal analysis, investigation. LY: data curation, investigation. DK: data curation, investigation, supervision. KJL: conceptualization, funding acquisition, methodology, project administration, resources, supervision, writing – review & editing.

## Funding support

This work is the result of NIH funding, in whole or in part, and is subject to the NIH Public Access Policy. Through acceptance of this federal funding, the NIH has been given a right to make the work publicly available in PubMed Central.

- National Institutes of Health, K99HL166861, ALK.
- Myocarditis Foundation, Postdoctoral Award P22-04488, ALK.
- National Institutes of Health, R01HL138466, KJL.
- National Institutes of Health, R01HL139714, KJL.
- National Institutes of Health, R01HL151078, KJL.
- National Institutes of Health, R01HL161185, KJL.
- National Institutes of Health, R35HL161185, KJL.
- Leducq Foundation, Network #20CVD02, KJL.
- Burroughs Welcome Fund, 1014782, KJL.
- Children’s Discovery Institute of Washington University and St. Louis Children’s Hospital, CH-II-2015-462, KJL.
- Children’s Discovery Institute of Washington University and St. Louis Children’s Hospital, CH-II-2017-628, KJL.
- Children’s Discovery Institute of Washington University and St. Louis Children’s Hospital, PM-LI-2019-829, KJL.
- Foundation of Barnes-Jewish Hospital, 8038-88, KJL.
- National Institutes of Health, Washington University in St. Louis Rheumatic Diseases Research Resource-Based Center Grant P30AR073752, KJL.
- Washington University School of Medicine support to KJL.

## Conflict of interest

The authors have declared that no conflict of interest exists.

## Supplementary Material

Supplemental data

Supplemental video 1

Supporting data values

## Version 1 07/07/2026

In-Press Preview

## Version 2 09/01/2026

Electronic publication
