# Supplementary material for: Fate-mapping infiltrating monocytes following experimental myocardial infarction reveals differentiation trajectories in the infarcted heart
Source: J Clin Invest. 2026 Jul 7;136(17):e189684. doi: 10.1172/JCI189684 (PMC13528930; doi:10.1172/JCI189684)
Supplement: Supplementary file 1 [file jci-136-189684-s313.pdf]

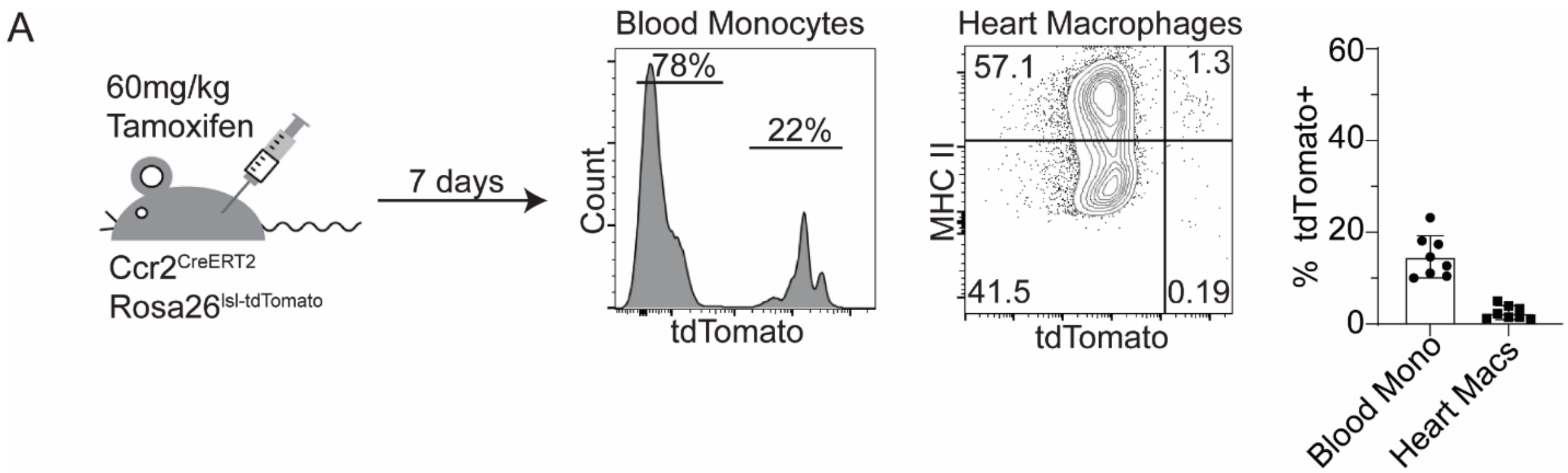

Suppl. Fig. 1 Single dose tamoxifen administration does not label resident macrophage in *Ccr2Cre^{ERT2}Rosa26^{tdTomato}* mice. A, Representative flow cytometry plots demonstrating that a single dose of tamoxifen administered to *Ccr2Cre^{ERT2}Rosa26^{tdTomato}* mice does not label resident cardiac macrophages in naïve mice 7 days after administration. Two tailed t-test with Welch's correction.

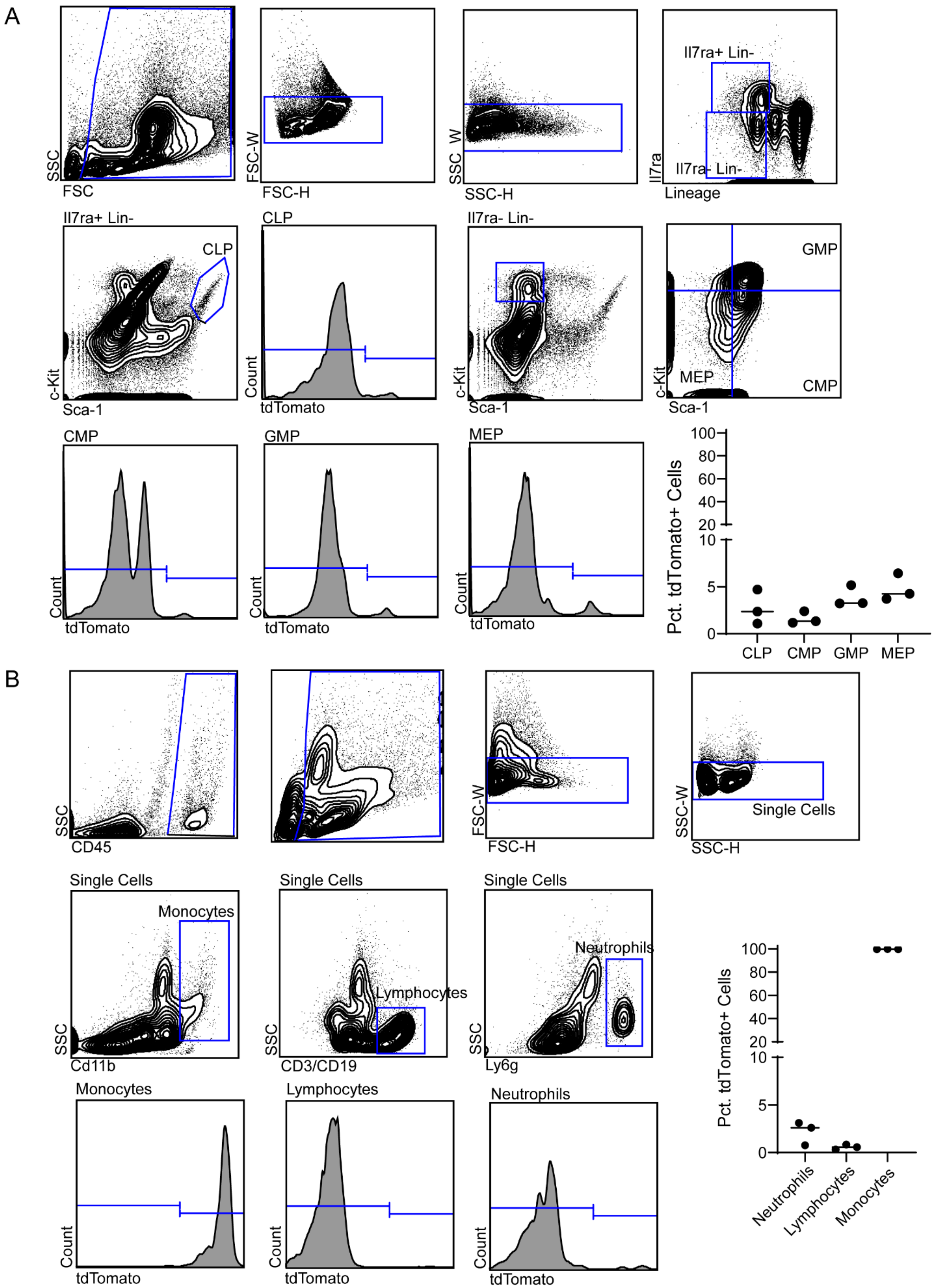
A
SSC
FSC
FSC-W
FSC-H
SSC W
SSC-H
Il7ra+ Lin-
Il7ra- Lin-
Il7ra
Lineage
Il7ra+ Lin-
CLP
c-Kit
Sca-1
CLP
Count
tdTomato
Il7ra- Lin-
c-Kit
Sca-1
GMP
MEP
CMP
c-Kit
Sca-1
CMP
Count
tdTomato
GMP
Count
tdTomato
MEP
Count
tdTomato
Pct. tdTomato+ Cells
100
80
60
40
20
10
5
0
CLP
CMP
GMP
MEP
B
SSC
CD45
FSC-W
FSC-H
SSC-W
SSC-H
Single Cells
Single Cells
Monocytes
SSC
Cd11b
Single Cells
Lymphocytes
SSC
CD3/CD19
Single Cells
Neutrophils
SSC
Ly6g
Monocytes
Count
tdTomato
Lymphocytes
Count
tdTomato
Neutrophils
Count
tdTomato
Pct. tdTomato+ Cells
100
80
60
40
20
10
5
0
Neutrophils
Lymphocytes
Monocytes

Suppl. Fig. 2 Bone marrow and blood FACS gating strategies. A, Representative gates and quantification of the percent of tdTomato positive progenitors cells in the bone marrow by flow cytometry in *Ccr2*^{CreERT2}*Rosa26*$^{LSL\text{-}tdTomato}$ mouse 1 day after tamoxifen injection. CLP: common lymphoid progenitor, CMP: common myeloid progenitor, GMP: granulocyte-monocyte progenitor, MEP: megakaryocyte-erythrocyte progenitor). **B**, Representative gates and quantification of the percent of tdTomato positive cells in the blood by flow cytometry in *Ccr2*^{CreERT2}*Rosa26*$^{LSL\text{-}tdTomato}$ mouse 1 day after tamoxifen.

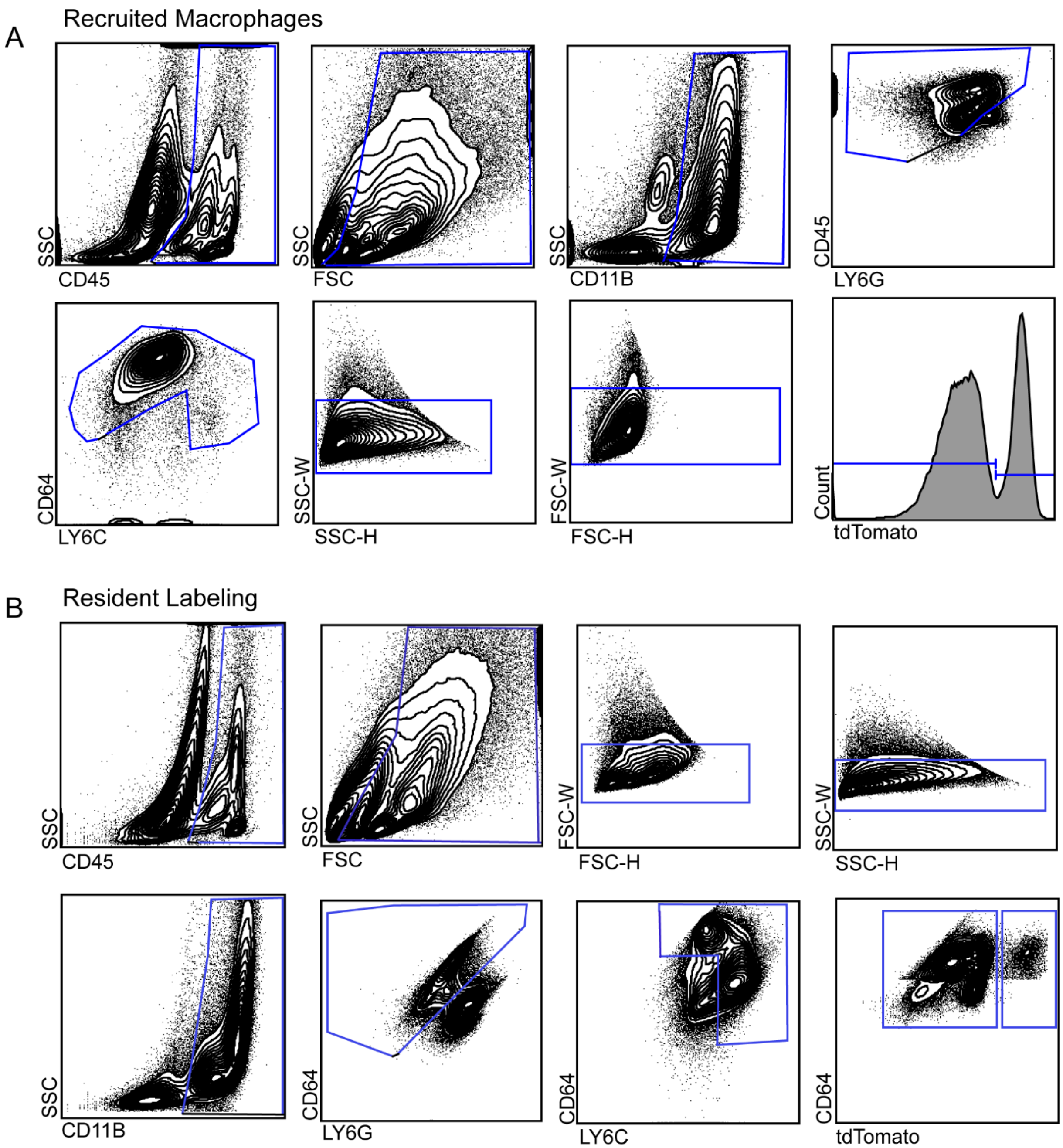

Suppl. Fig. 3 FACS gating strategies for constructing monocyte and cardiac resident macrophage single cell RNA sequencing libraries. A, Flow cytometry gating strategies used to isolate cardiac monocytes and macrophages for monocyte lineage tracing single cell RNA sequencing libraries. **B**, Flow cytometry gating strategies used to isolate cardiac monocytes and macrophages for cardiac resident macrophage lineage tracing single cell RNA sequencing libraries.

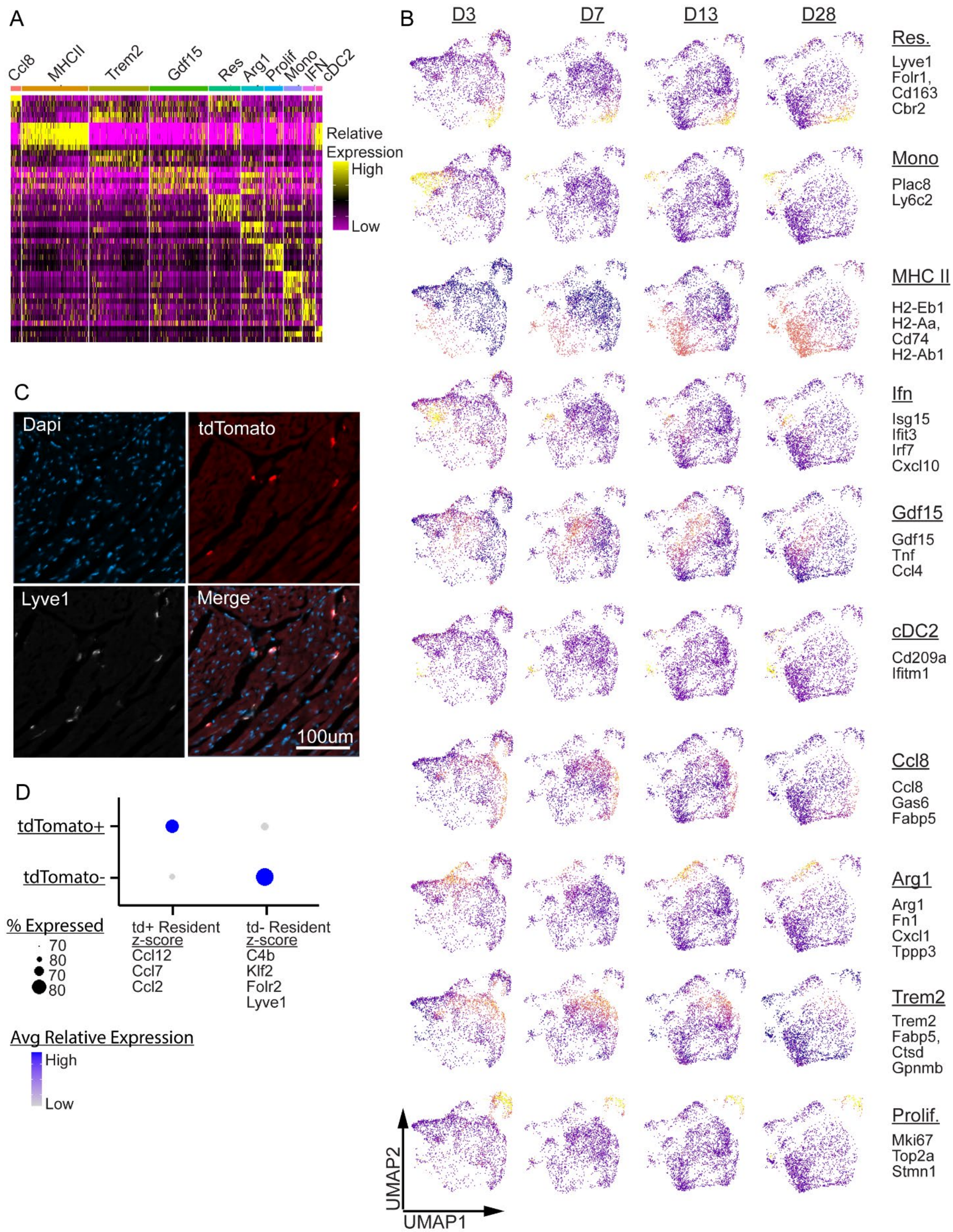
A
Ccl8
MHCII
Trem2
Gdf15
Res
Arg1
Prolif
Mono
IFN
cDC2
Relative Expression
High
Low
B
D3
D7
D13
D28
Res.
Lyve1
Folr1,
Cd163
Cbr2
Mono
Plac8
Ly6c2
MHC II
H2-Eb1
H2-Aa,
Cd74
H2-Ab1
Ifn
Isg15
Ifit3
Irf7
Cxcl10
Gdf15
Gdf15
Tnf
Ccl4
cDC2
Cd209a
Ifitm1
Ccl8
Ccl8
Gas6
Fabp5
Arg1
Arg1
Fn1
Cxcl1
Tppp3
Trem2
Trem2
Fabp5,
Ctsd
Gpnmb
Prolif.
Mki67
Top2a
Stmn1
UMAP2
UMAP1
C
Dapi
tdTomato
Lyve1
Merge
100um
D
tdTomato+
tdTomato-
% Expressed
70
80
70
80
td+ Resident
z-score
Ccl12
Ccl7
Ccl2
td- Resident
z-score
C4b
Klf2
Folr2
Lyve1
Avg Relative Expression
High
Low

Suppl. Fig. 4 Single cell RNA sequencing of recruited monocytes and their progeny after MI. A, Heatmap of top 5 enriched genes in each monocyte, macrophage, and dendritic cell cluster. **B**, UMAP projections of marker genes (expressed as z-scores) for each cluster split by time point post-MI. **C**, Representative immunostaining image of LYVE1 expression in tdTomato+ macrophage in $Ccr2^{CreERT2}Rosa26^{lsl\text{-}tdTomato}$ hearts 4 weeks after MI. **D**, Dot plot of transcriptional signatures that differentiate tdTomato- resident cardiac macrophages and tdTomato+ resident-like cardiac macrophages.

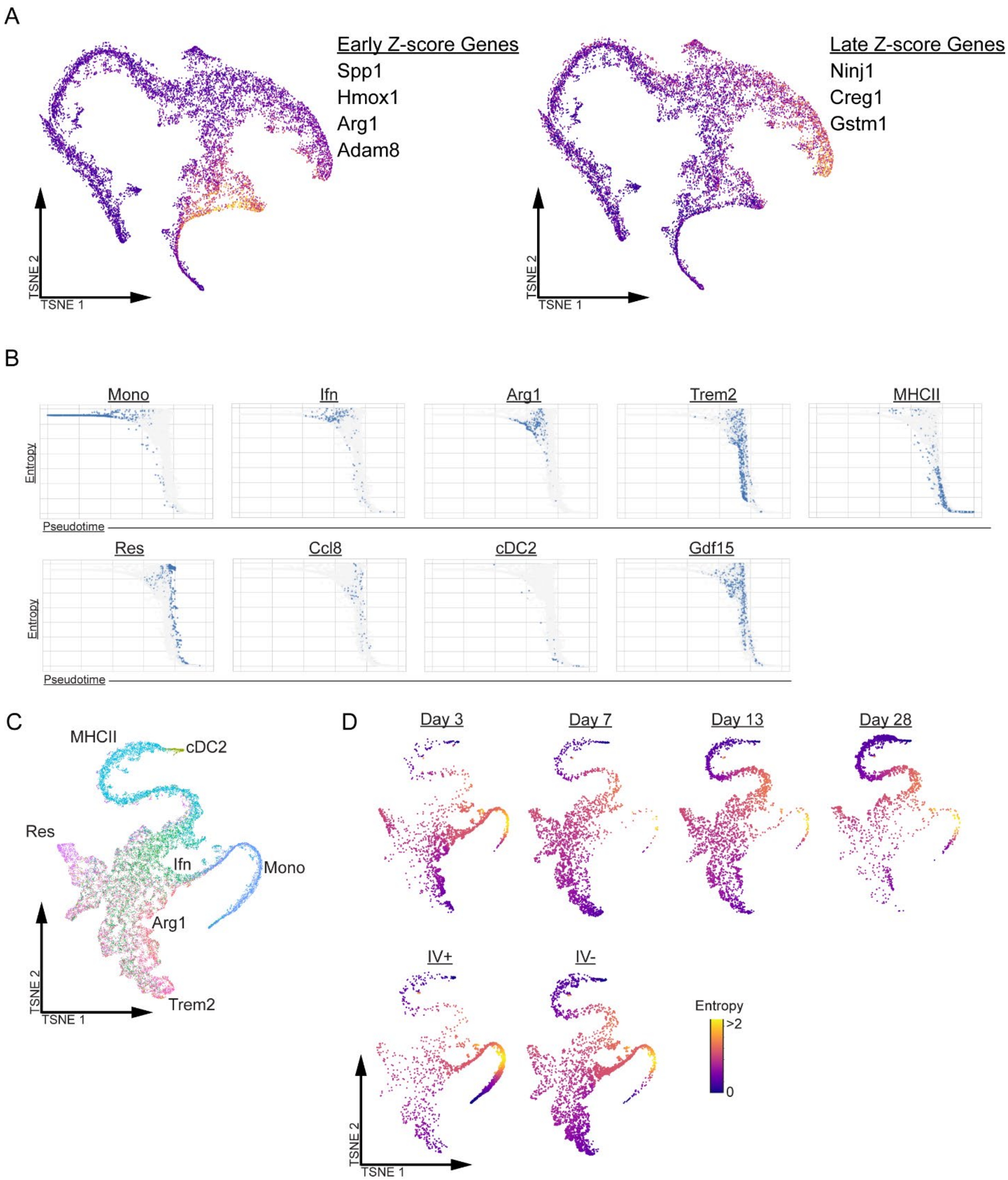

Suppl. Fig. 5 Monocyte differentiation trajectories predicted by pseudotime analysis. A, tSNE feature plot of transcriptional signatures (displayed as a z-score) enriched in the early (left) and late (right) *Trem2*+ macrophage populations. **B**, Waterfall plots of pseudotime versus differentiation potential split by cluster. **C**, tSNE plots split by condition (lineage tracing and anti-CD45 antibody IV labeling) showing entropy values.

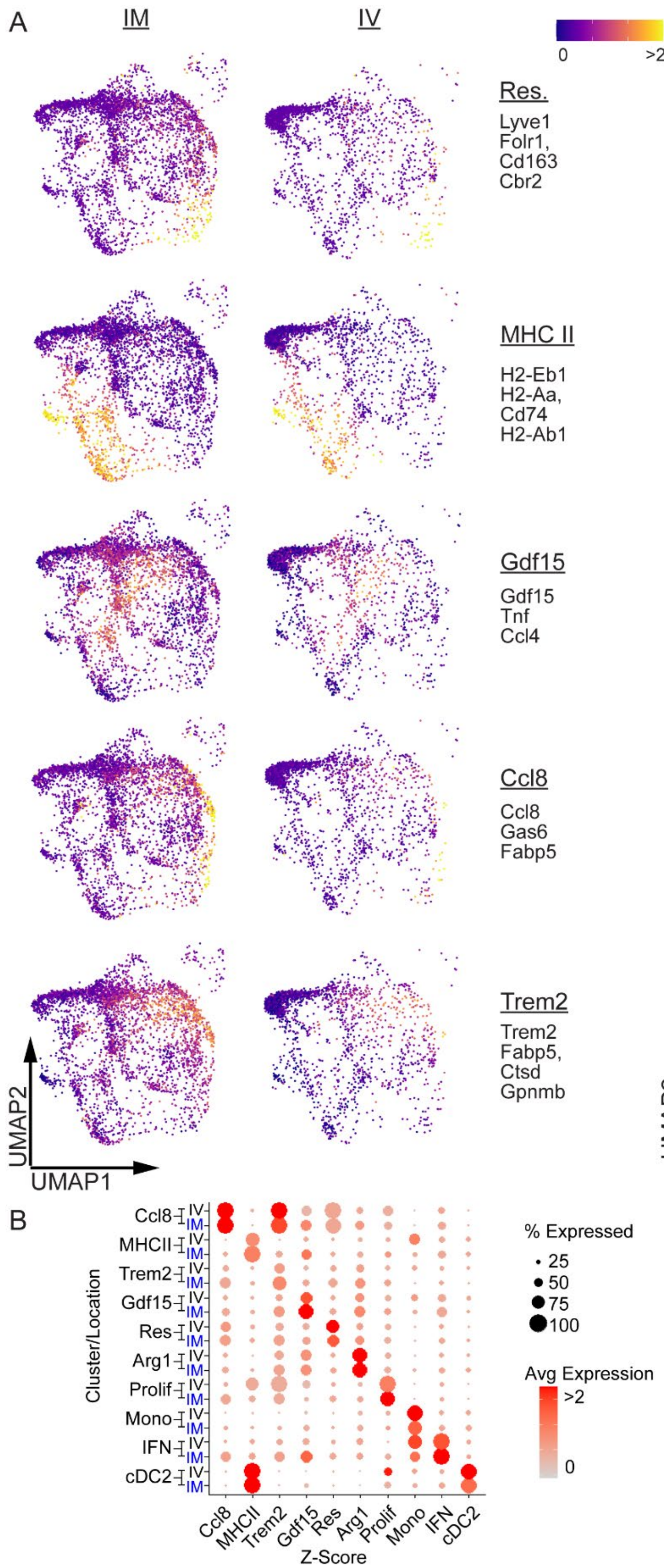

A
IM
IV
0
>2
IM
IV
Res.
Lyve1
Folr1,
Cd163
Cbr2
Mono
Plac8
Ly6c2
MHC II
H2-Eb1
H2-Aa,
Cd74
H2-Ab1
Ifn
Isg15
Ifit3
Irf7
Cxcl10
Gdf15
Gdf15
Tnf
Ccl4
cDC2
Cd209a
Ifitm1
Ccl8
Ccl8
Gas6
Fabp5
Arg1
Arg1
Fn1
Cxcl1
Tppp3
Trem2
Trem2
Fabp5,
Ctsd
Gpnmb
Prolif.
Mki67
Top2a
Stmn1
UMAP2
UMAP1
UMAP2
UMAP1
B
Cluster/Location
Ccl8
MHCII
Trem2
Gdf15
Res
Arg1
Prolif
Mono
IFN
cDC2
IV
IM
% Expressed
25
50
75
100
Avg Expression
>2
0
Ccl8
MHCII
Trem2
Gdf15
Res
Arg1
Prolif
Mono
IFN
cDC2
Z-Score

Suppl. Fig. 6 Single cell RNA sequencing of intravascular and extravascular monocytes and macrophages after MI. A, UMAP projections of genes (displayed as z-scores) enriched in each cluster split by intravascular vs. extravascular compartment. **B**, Dot plots of marker gene z-scores in each cluster split by intravascular vs. extravascular compartment.

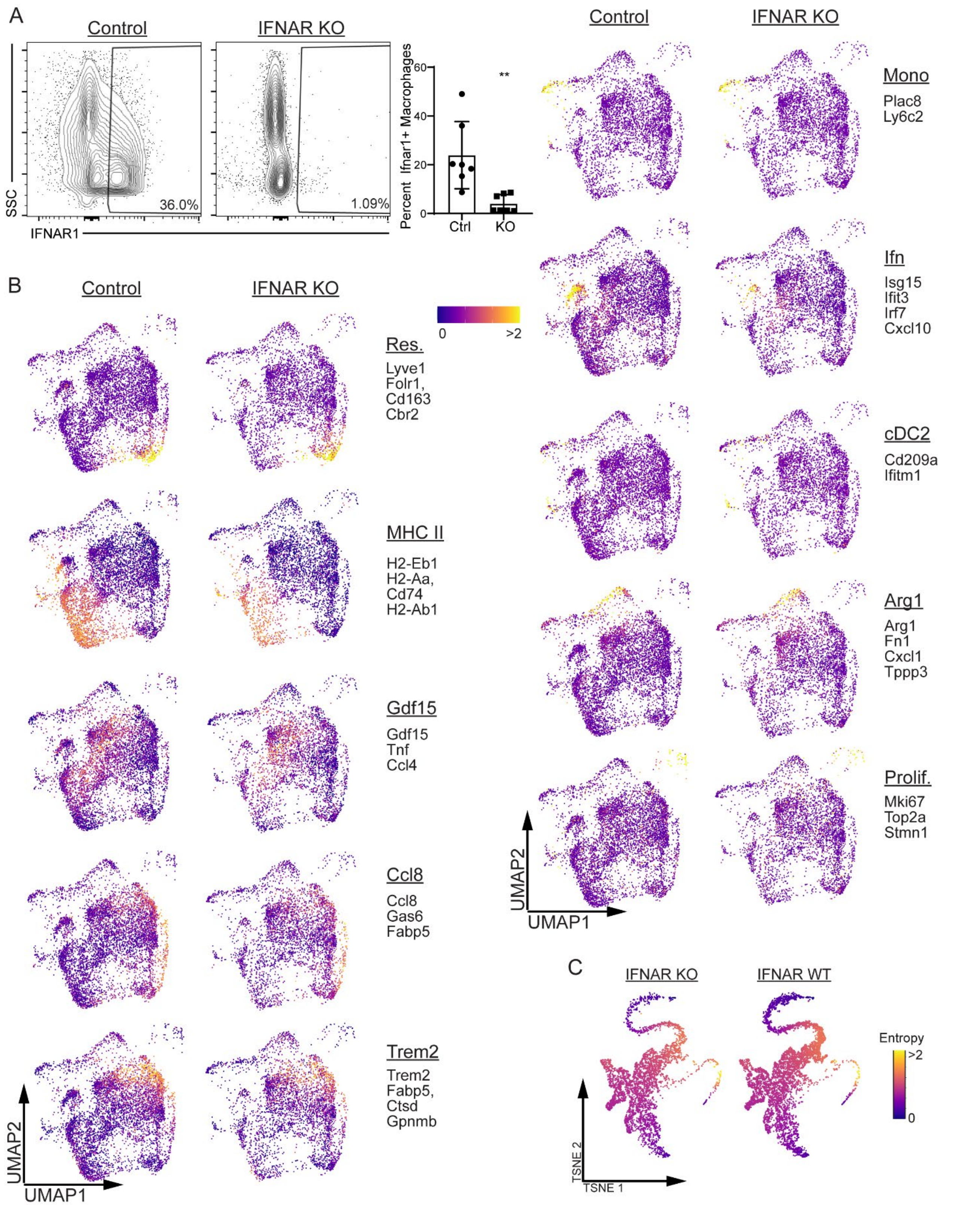
A
Control
IFNAR KO
SSC
IFNAR1
36.0%
1.09%
Percent Ifnar1+ Macrophages
60
40
20
0
**
Ctrl
KO
B
Control
IFNAR KO
0
>2
Res.
Lyve1
Folr1,
Cd163
Cbr2
MHC II
H2-Eb1
H2-Aa,
Cd74
H2-Ab1
Gdf15
Gdf15
Tnf
Ccl4
Ccl8
Ccl8
Gas6
Fabp5
Trem2
Trem2
Fabp5,
Ctsd
Gpnmb
UMAP2
UMAP1
Control
IFNAR KO
Mono
Plac8
Ly6c2
Ifn
Isg15
Ifit3
Irf7
Cxcl10
cDC2
Cd209a
Ifitm1
Arg1
Arg1
Fn1
Cxcl1
Tppp3
Prolif.
Mki67
Top2a
Stmn1
UMAP2
UMAP1
C
IFNAR KO
IFNAR WT
Entropy
>2
0
TSNE 2
TSNE 1

Suppl. Fig. 7 Single cell RNA sequencing of monocytes and macrophages from control and *Ccr2*^{ERT2Cre}*Ifnar*^{Flox} hearts. A, Representative flow cytometry plots and quantification of IFNAR1 expression in induced peritoneal macrophages. Two tailed t-test with Welch's correction. **B**, UMAP projections of genes enriched (displayed as z-scores) in each cluster split by experimental condition. **C**, tSNE plots showing entropy values split by experimental condition.

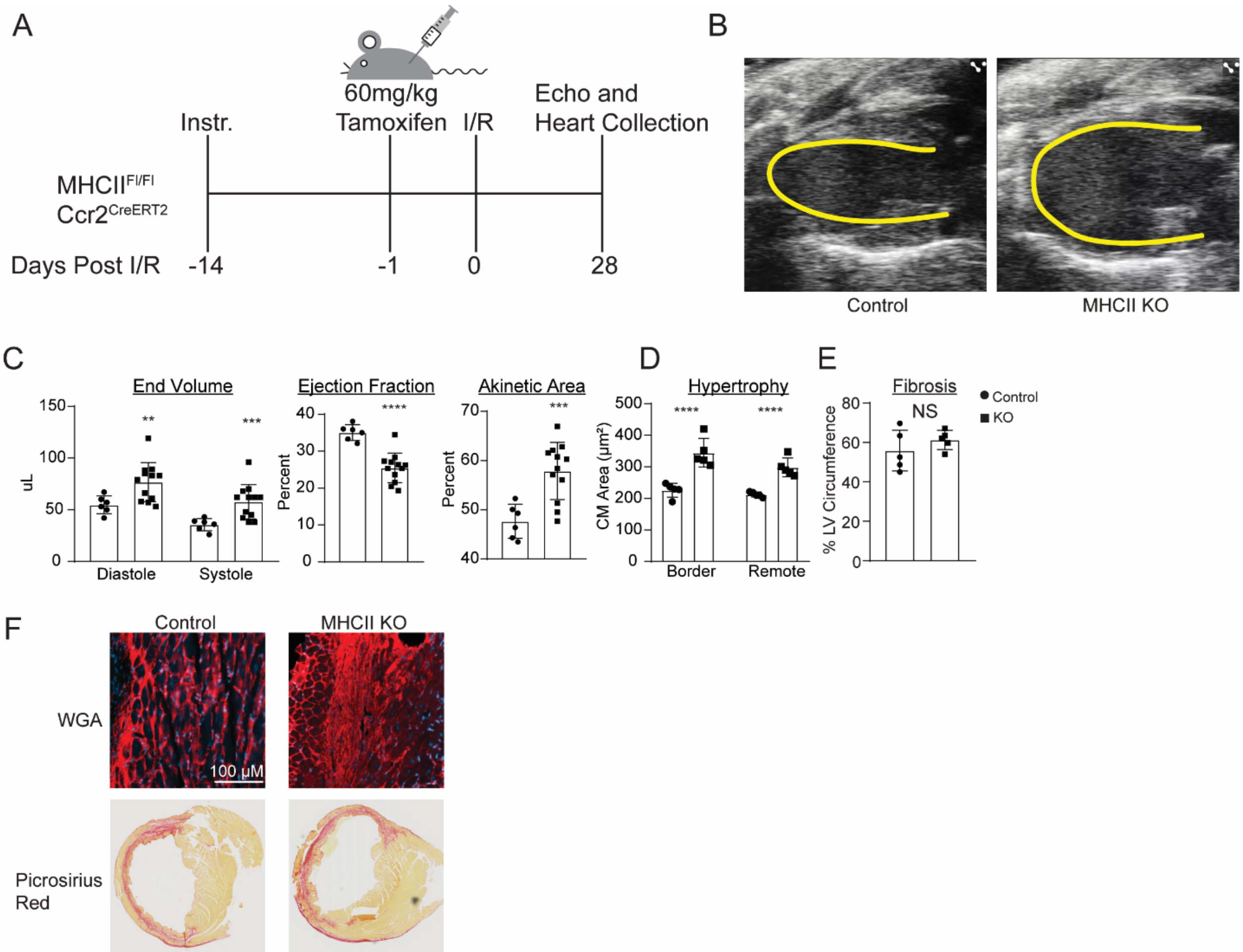

Suppl. Fig. 8 MHCII^{hi} macrophages are protective in myocardial infarction. A, Experimental strategy for deletion of *MHCII* in recruited monocytes and their progeny. **B**, Representative long-axis echocardiographic images at end diastole showing enhanced LV remodeling in *Ccr2^{ERT2Cre}Ab1^{Flox}* mice compared to controls. **C**, Quantification of echocardiography measurements of LV end systolic volume, ejection fraction, and akinetic area 4 weeks after MI. Two tailed t-test with Welch's correction. **D**, Measurement of cardiomyocyte size by WGA staining 4 weeks after MI. Two tailed t-test with Welch's correction. **E**, Measurement of infarct size assessed by fibrosis as a percentage of LV diameter 4 weeks after MI. Two tailed t-test with Welch's correction. **F**, Representative images of WGA staining at the border zone and trichrome staining in control and *Ccr2^{ERT2Cre}Ifnar^{Flox}* hearts.

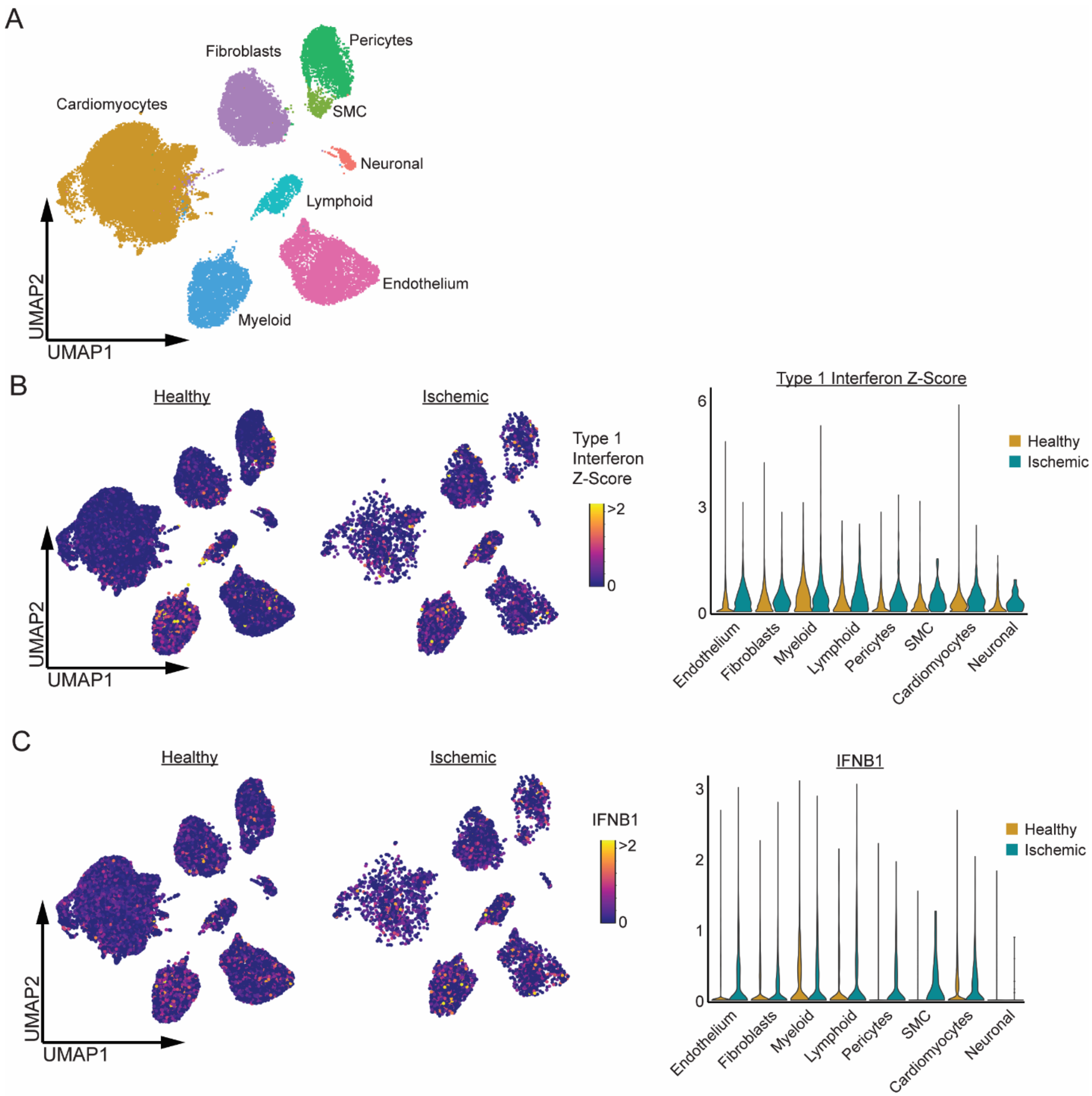

Suppl. Fig. 9 Type 1 Interferon is produced by multiple cell types in ischemic cardiac injury. A, UMAP projection of single nuclei RNA sequencing of ischemic and healthy human cardiac tissue. **B,** UMAP feature plot and violin plot of Type 1 interferon ligand z-score across cell types and conditions. **C,** UMAP feature plot and violin plot of IFNB1 across cell types and conditions.
